# Mechanisms of Groucho-mediated repression revealed by genome-wide analysis of Groucho binding and activity

**DOI:** 10.1186/s12864-017-3589-6

**Published:** 2017-02-28

**Authors:** Michael Chambers, Wiam Turki-Judeh, Min Woo Kim, Kenny Chen, Sean D. Gallaher, Albert J. Courey

**Affiliations:** 1 Department of Chemistry and Biochemistry, University of California, Los Angeles, CA 90095 USA; 2 Molecular Biology Institute, University of California, Los Angeles, CA 90095 USA; 3 Department of Energy, Institute of Genomics and Proteomics, University of California, Los Angeles, CA 90095 USA

**Keywords:** Groucho, Transcriptional repression, Drosophila embryogenesis, ChIP-seq, RNA-seq, Chromatin-associated RNA-seq, RNA polymerase II pausing

## Abstract

**Background:**

The transcriptional corepressor Groucho (Gro) is required for the function of many developmentally regulated DNA binding repressors, thus helping to define the gene expression profile of each cell during development. The ability of Gro to repress transcription at a distance together with its ability to oligomerize and bind to histones has led to the suggestion that Gro may spread along chromatin. However, much is unknown about the mechanism of Gro-mediated repression and about the dynamics of Gro targeting.

**Results:**

Our chromatin immunoprecipitation sequencing analysis of temporally staged Drosophila embryos shows that Gro binds in a highly dynamic manner primarily to clusters of discrete (<1 kb) segments. Consistent with the idea that Gro may facilitate communication between silencers and promoters, Gro binding is enriched at both cis-regulatory modules, as well as within the promotors of potential target genes. While this Gro-recruitment is required for repression, our data show that it is not sufficient for repression. Integration of Gro binding data with transcriptomic analysis suggests that, contrary to what has been observed for another Gro family member, Drosophila Gro is probably a dedicated repressor. This analysis also allows us to define a set of high confidence Gro repression targets. Using publically available data regarding the physical and genetic interactions between these targets, we are able to place them in the regulatory network controlling development. Through analysis of chromatin associated pre-mRNA levels at these targets, we find that genes regulated by Gro in the embryo are enriched for characteristics of promoter proximal paused RNA polymerase II.

**Conclusions:**

Our findings are inconsistent with a one-dimensional spreading model for long-range repression and suggest that Gro-mediated repression must be regulated at a post-recruitment step. They also show that Gro is likely a dedicated repressor that sits at a prominent highly interconnected regulatory hub in the developmental network. Furthermore, our findings suggest a role for RNA polymerase II pausing in Gro-mediated repression.

**Electronic supplementary material:**

The online version of this article (doi:10.1186/s12864-017-3589-6) contains supplementary material, which is available to authorized users.

## Background

The Groucho (Gro)/Transducin-like enhancer of split (TLE) family of corepressors plays crucial roles in the interpretation and integration of multiple spatially and temporally regulated cell intrinsic and extrinsic inputs during metazoan development, thus helping to generate precisely regulated spatial and temporal patterns of gene expression [1]. Drosophila Gro is recruited to genomic loci through interactions with a diverse array of transcriptional repressors [2]; through these interactions, it is essential for nearly all aspects of embryonic and imaginal development [1, 3]. In humans, Gro/TLE family proteins are involved in such processes as organ development, adipogenesis, neurogenesis, hematopoiesis, and osteogenesis [4–7].

Gro can repress transcription even when recruited to sites thousands of basepairs away from a core promoter and/or from the activation elements acting on a promoter [1, 8]. While the mechanism of long-range Gro-mediated repression is not clearly understood, one possibility is that Gro spreads along chromatin fibers to generate large transcriptionally silent domains. Support for this model comes from the following observations: (1) Gro forms tetramers and higher order oligomers and repression can be compromised by mutations that prevent oligomerization [9–13]; (2) Gro recruits the histone deacetylase Rpd3 resulting in histone hypoacetylation and Gro function can be compromised by Rpd3 mutations and by histone deacetylase inhibitors [14–18]; and (3) Gro binds to hypoacetylated histone tails [19, 20]. Thus, in a mechanism analogous to that proposed for repression in budding yeast by Sir family corepressors [8, 21], initial recruitment of Gro may lead to histone deacetylation by Rpd3, the recruitment of additional Gro through interactions with the deacetylated histones, and propagation of Gro along chromatin facilitated by Gro self-association. However, recent evidence in cell culture has shown that Gro binds in discrete peaks, although longer stretches of binding do occur [22]. Additionally, loss of the ability to oligomerize failed to decrease median peak widths significantly, although it did result in the identification of fewer Gro-associated regions [22].

To further elucidate the mechanisms and targets of Gro-mediated repression, we have conducted a genome-wide analysis of Gro binding and repression at multiple stages of *Drosophila* embryonic development. We find that Gro associates with chromatin in discrete usually transient peaks often clustered upstream of or within regulated genes in a pattern that is not compatible with a simple spreading model for long-range repression. By combining genome-wide chromatin binding and gene expression analysis, we have also identified a set of high-confidence Gro targets, allowing more confident positioning of Gro within the developmentally-regulated gene network. These high confidence targets are highly enriched for promoter-proximal paused RNA polymerase II (Pol II), suggesting a role for Pol II pausing in Gro-mediated repression.

## Methods

### Fly strains

Flies were maintained on standard medium at 25 °C. UAS-*Gro* transgenic flies were described previously [23]. Embryos for overexpression studies were obtained from staged embryos collected from crosses of UAS-*Gro* with a maternal driver, *Mat-Gal4* [23]. Control embryos for RNA sequencing (RNA-seq) were obtained from crossing *w* ^*1118*^ flies with this *Mat-Gal4* driver. Germ line clones of the *gro* mutant fly allele MB36 (a null allele) were used for Groucho loss-of-function studies [24]. These clones were generated using the standard dominant female sterile FLP/FRT protocol [25].

### Groucho chromatin immunoprecipitation (ChIP) and sequencing

Chromatin immunoprecipitation (ChIP) was carried out as described previously [26]. Embryos were collected in three successive 2.5 h windows beginning 1.5 h post-deposition from OregonR population cages and crosslinked with formaldehyde prior to sonication (Diagenode Bioruptor). Immunoprecipitation was carried out using rabbit polyclonal antibodies raised against the Gro-GP domain GST fusion protein affinity purified against the Halo-tagged GP domain. Libraries for multiplex sequencing were prepared using the Nugen Ovation Ultralow System V2 kit (catalog # 0344–32).

### Groucho ChIP sequencing (ChIP-seq) data analysis

Multiplexed libraries were sequenced on Illumina HiSeq 2000 sequencing platforms (High Throughput Sequencing Facility, Broad Stem Cell Research Center, UCLA). Reads were demultiplexed via custom scripts. Demultiplexed libraries were filtered for read quality and PCR duplicates. The number of non-redundant mapped reads varied from ~7.1 million to ~9.3 million for the six experiments (two from each of three timepoints (Additional file 1: Table S1)). Alignment was performed against the *Drosophila melanogaster* genome (iGenomes BDGP 5.25 assembly) with Bowtie2 (v2.2.5) using the following parameters: −*very-sensitive-local* [27]. Peak calling was performed using MACS2 (v2.1.0) with default parameters [28]. Peak visualizations were generated with Integrated Genome Browser (v8.4.2) [29]. Peaks with a minimum 1 bp overlap between replicates were used for further analysis, unless otherwise noted (ChIPpeakAnno) [30]. Motif enrichment analysis was performed with the DREME software suite (v4.10.1) on 500 base pair regions centered on ChIP-seq peaks identified by MACS2 [31].

### Embryonic polyA(+) RNA isolation and sequencing

Wild-type and mutant embryos were collected in three successive 2.5 h windows beginning 1.5 h post-deposition. Embryos were manually homogenized in TRIzol reagent (Invitrogen) and RNA was extracted according to the manufacturer’s protocol. Purified RNA quality was assessed on a Bioanalyzer 2100 (Agilent Technologies). Strand-specific polyA-selected libraries were generated with the TruSeq Stranded mRNA Library Prep Kit (Illumina) and sequenced on the Illumina HiSeq 2000 platform.

### Transcriptome (RNA-seq) data preparation and genomic alignment

Reads were demultiplexed via custom scripts. Low quality reads were trimmed and remaining reads were aligned with TopHat2 (v2.0.9) [32] against the *Drosophila melanogaster* genome (iGenomes BDGP 5.25 assembly) with iGenomes gene models as a guide. Assignment of reads to genes and calculation of genewise read counts were performed with HTSeq [33]. We obtained at least 19 million uniquely mapped reads for each of 24 experiments (Additional file 1: Table S1).

### Gene expression and Groucho target gene identification

Normalization of gene expression values and differential expression analysis were performed with DESeq2 (v1.8.0) [34]. Genes exhibiting a log_2_(fold-change) of magnitude 0.5 or greater with a corrected *p*-value of < 0.05 were called as significantly differentially expressed.

Gro occupancy scores were calculated using a modified version of a previously published scoring algorithm [35]. For each gene, a Gro occupancy score was calculated as the sum of the scores of Gro peaks. Scores for each peak were calculated on a per-base level and averaged. For each basepair overlapping the gene, a score of 1 was assigned. For each non-overlapping basepair, the score was calculated by

$$ \frac{1}{1 + {e}^{0.0005*\left( d-15\right)}} $$

where *d* is the distance between the basepair and the nearest end of the gene.

### Chromatin-associated RNA isolation from embryos and rRNA depletion

Wild-type (OregonR) fly embryos were collected in three successive 2.5 h windows beginning 1.5 h post-deposition. Between 3 and 5 g of embryos were utilized for each fractionation. The chromatin-associated RNA isolation protocol was adapted from previously described procedures [36, 37]. Embryos were dechorionated in 50% bleach for 90 s and transferred to a chilled Dounce homogenizer. Embryos were then rinsed three times with 25 ml of homogenization buffer (15 mM HEPES-KOH pH 7.6; 10 mM KCl; 3 mM CaCl_2_; 2 mM MgCl_2_; 0.1% Triton X-100; 1 mM DTT; 0.1 mM PMSF; 0.1x RNase inhibitor [RNasin, Promega]). Embryos were then suspended in homogenization buffer containing 0.3 M (15 ml) sucrose and dounced five times each with loose and tight pestles. Embryo lysate was filtered through 50-micron nylon cell strainer. Clarified lysate was layered over a sucrose cushion consisting of a layer of 1.7 M sucrose (15 ml) underneath a layer of 0.8 M sucrose (15 ml) in homogenization buffer. The samples were centrifuged at 15,000 RCF for 10 min at 4 °C. Pelleted nuclei were resuspended in 250 μl of nuclear lysis buffer (10 mM HEPES-KOH pH 7.6; 100 mM KCl; 0.1 mM EDTA; 10% glycerol; 0.15 mM spermine; 0.5 mM spermidine; 0.1 mM NaF; 0.1 mM Na_3_VO_4_; 0.1 mM ZnCl_2_; 1 mM DTT; 0.1 mM PMSF; 1x RNase inhibitor). While gently vortexing, an equal volume of NUN buffer (25 mM HEPES-KOH pH 7.6; 300 mM NaCl; 1 M urea; 1% NP-40; 1 mM DTT; 0.1 mM PMSF) was added drop-by-drop over a period 5 min. Condensed chromatin became visible as a fluffy white precipitate. The solution was then incubated for 20 min on ice and centrifuged at 14,000 rpm for 30 min at 4 °C. The supernatant (primarily nucleoplasm) was discarded and the pellet was resuspended in Trizol reagent (Qiagen). RNA was then purified following the manufacturer’s protocol.

RNA samples were depleted of ribosomal, poly(A)+, and additional RNA contaminants through an affinity depletion procedure adopted from a published protocol [37]. An equimolar mixture of biotinylated affinity oligomers (Additional file 2: Table S2; Eurofins MWG Operon) was added to 6 μg of purified RNA in annealing buffer (10 mM EDTA; 0.5x SSC) in a volume of 100 μl. RNA was denatured at 75 °C for 5 min and annealed at 37 °C for 30 min. The annealed mixture was added to 1 ml streptavidin paramagnetic beads (Promega) and incubated at 25 °C for 15 min, followed by 2 h at 4 °C with gentle rocking, and the supernatant retained for library preparation. This procedure was performed twice per sample.

### Chromatin-associated RNA-seq library construction and sequencing

rRNA-depleted RNA was concentrated via ethanol precipitation. Size distribution of samples was determined via Agilent 2100 Bioanalyzer (Agilent Technologies). Indexed RNA-seq libraries were generated with the ScriptSeq v2 RNA-seq Library Preparation Kit (Epicentre). Sequencing was performed on the Illumia HiSeq 2000 sequencing platform (High Throughput Sequencing Core Facility, Broad Stem Cell Research Center, UCLA). Reads were demultiplexed and mapped as described above for poly(A) + RNA. We obtained at least 13 million reads per replicate (Additional file 1: Table S1).

### Chromatin-associated RNA-seq data analysis

Mean normalized transcript expression levels (FPKM) were generated with DESeq2 (v1.10.0) [34]. Significant changes in transcript abundance were quantified with DESeq2 by comparison with poly(A) + RNA-seq from wild-type embryo data described above. RNA-seq read mapping density analysis was performed using PicardTools (http://broadinstitute.github.io/picard/). Additional metagene analysis was performed using the ‘metagene’ package of R/Bioconductor [38].

## Results

### Gro is transiently recruited to thousands of sites in the developing embryo

Using a validated affinity purified polyclonal antibody raised against the Gro GP domain (Additional file 3: Figure S1A), Gro chromatin immunoprecipitation sequencing (ChIP-seq) was performed on fly embryos collected during three successive 2.5 h timespans collectively encompassing 1.5 to 9 h of development. Libraries were sequenced to a depth that provided at minimum 5 million uniquely mappable reads, well in excess of the minimum recommended by modENCODE ChIP-seq best-practices (Additional file 1: Table S2) [39]. Replicates exhibited high reproducibility (Additional file 3: Figure S1B, C).

The high degree of correlation between our ChIP-seq data sets and the modENCODE ChIP-chip data sets obtained from 0–12 h embryos [40] using completely independent antibodies also validates our ChIP-seq data (Additional file 3: Figure S1D). The modENCODE Gro peaks were generated from 0–12 h embryos and so should represent a time-averaged superset of our data. Collectively the ChIP-seq peaks from our three time points identified 79% of the modENCODE ChIP-chip peaks. However, 81% of our identified Gro binding sites are not represented in the data generated by the modENCODE consortium. This probably reflects the greater sensitivity of ChIP-seq compared to ChIP-chip as well as the use of more narrowly staged embryos, which may increase the signal-to-noise ratio. Comparison of our ChIP-seq data with modENCODE Gro ChIP-chip data generated from white pre-pupae also shows a significant overlap (Additional file 3: Figure S1E). About a third of embryonic peaks are retained in this later stage, indicating that Gro may be utilized in the regulation of a subset of common genes throughout multiple developmental stages. Nonetheless, a large fraction of embryonic and pre-pupal binding sites are unique to each stage, consistent with the distinct roles of Gro-mediated repression during pupal development [41].

**Fig. 1:**
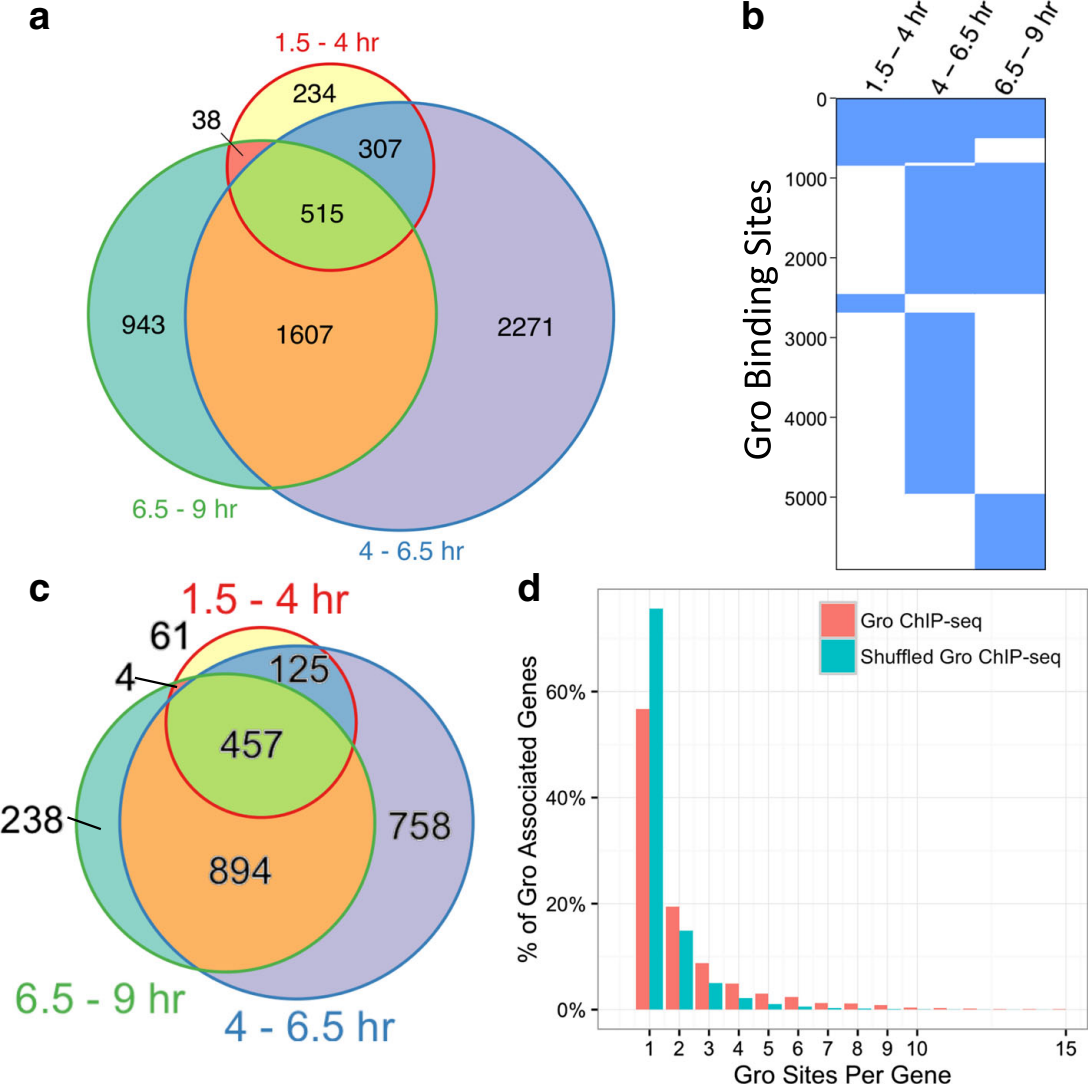
Gro binding is highly dynamic. **a** Analysis of Gro binding sites: Gro ChIP-seq was carried out in duplicate on 1.5–4 h, 4–6.5 h, and 6.5–9 h embryo collections. Putative binding sites (ChIP-seq peaks) were identified as described in Materials and Methods. The Venn diagram indicates overlap between binding sites at the three time points analyzed. **b** Clustering of Gro binding sites by temporal pattern. The majority of Gro binding sites are unique to a single time point, while many were observed at all three time points. Although a substantial fraction of sites overlap between time points 2 and 3, very few overlap between time points 1 and 2. A small number of sites (38) are bound in only time points 1 and 3 without being bound in 2, indicating that loss of Gro from a locus tends to be a permanent regulatory decision. **c** Analysis of Gro-bound genes: Each Gro binding site was assigned to the closest gene. The Venn diagram indicates overlap between Gro bound genes at the three time points analyzed. **d** Distribution of the number of Gro binding sites per gene: About 45% of all Gro-bound genes exhibit two or more distinct Gro binding sites peaks, a fraction that is greater than that expected by chance (*p* < 10^−10^ by Monte Carlo simulation)

Peak modeling identified widespread Gro binding throughout the genome; peaks observed in both replicates were chosen for further analysis, as they represent a higher confidence subset of all identified peaks. Gro binding regions are most numerous during time point 2 (4–6.5 h of development; ~4,700 binding sites), compared to the time point 1 (1.5–4 h; ~1,100 binding sites) and time point 3 (6.5–9 h; ~3,100 binding sites). Gro occupancy is highly dynamic and reversible. Approximately 58% of all Gro binding sites are unique to a single time point, while only about 9% of Gro binding sites are occupied constitutively throughout the time span analyzed (Fig. 1a, b).

**Fig. 2:**
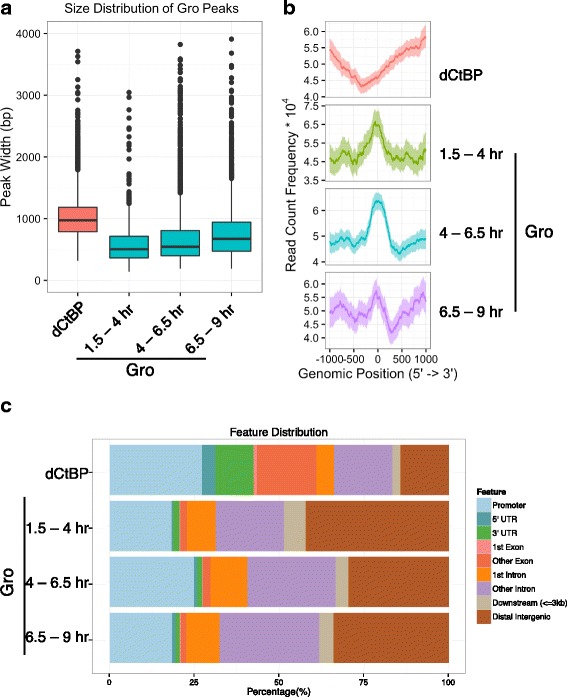
The pattern of Groucho recruitment to chromatin differs from that of the corepressor dCtBP. **a** Distribution of Gro and dCtBP binding site size. As indicated by a box plot, Groucho sites exhibit median widths of between 500 and 750 basepairs at the three time points sampled, although significantly larger peaks were also identified. dCtBP binding sites exhibits a median width of about 1000 bp. Thus, the difference between the short-range repression mediated by dCtBP and the long-range repression mediated by Gro cannot be attributed to one-dimensional spreading along chromatin. **b** Distribution of Gro and dCtBP binding sites relative to the nearest transcriptional start site (TSS). Gro ChIP-seq read density is enriched around TSS’s compared to dCtBP, which exhibits a small asymmetric depletion in these areas. **c** Distribution of Gro and dCtBP binding sites with respect to gene feature. Compared to dCtBP, Gro more often binds in intergenic regions, and therefore less frequently within either intronic or exonic regions

Choosing the nearest or overlapping gene as a potential Gro-regulated target, we observed significantly fewer Gro-associated genes than Gro binding regions (Fig. 1c) due to the tendency of Gro to localize to multiple discrete regions around its potential targets. Half of all Gro-associated genes predicted in this fashion have two or more Gro peaks in relative proximity (Fig. 1d), with an average of 2.5 binding sites per associated gene (compared to an expected value of 1.5 binding sites per gene given a random distribution of Gro peaks, *p* < 10^−10^ via Monte-Carlo simulation). These peaks have median widths in the 500–700 bp range (Fig. 2a), indicative of point source peaks, as commonly seen for sequence-specific transcription factors [42], rather than the broad peaks typical of either highly polymeric factors or histone marks. Consistent with this finding regarding Gro peak width, in vitro studies have shown that Grg3, a mammalian Gro homolog, binds and protects DNA from nucleases over a span of 3 to 4 nucleosomes [43], corresponding to 600–800 basepairs of protection.

dCtBP is a short-range corepressor known to interact with multiple repressors, including Brinker, Snail, and Knirps [44, 45]. Therefore, we compared patterns of Gro and dCtBP binding in the embryo. dCtBP binding sites were obtained from published ChIP-chip data on 0–12 hr embryos [40]. We find that only between 8 and 10% of Gro binding sites overlap dCtBP sites, and that, despite sharing multiple interacting partners, the two corepressors exhibit distinct patterns of genomic recruitment, with dCtBP more likely to be recruited within gene bodies (Fig. 2c). Gro is more often recruited within intergenic regions, but is also enriched for binding within promoter regions, where dCtBP is slightly depleted (Fig. 2b). These findings suggest that Gro and dCtBP employ distinct mechanisms to repress transcription and is consistent with the notion that they direct long- and short-range repression, respectively. However, the two factors exhibit similar size binding sites (Fig. 2a), arguing against the possibility that one-dimensional Gro spreading is responsible for the longer-range repression generally associated with Gro.

### Gro is recruited to sites of Dorsal-mediated activation and repression

**Fig. 3:**
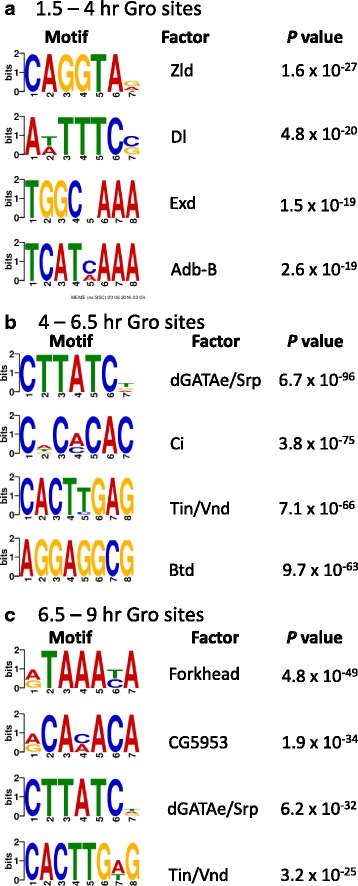
Gro binding sites are enriched for binding motifs of multiple sequence-specific transcription factors. The four most significantly enriched sites as identified by *de novo* motif discovery (DREME) are shown for each timepoint. For each motif, the factor with the most similar binding site is listed. In cases where the discovered motif corresponds with similar likelihoods to binding sites for multiple factors, all potential factors are listed. **a** Motifs enriched in 1.5–4 h Gro binding sites. **b** Motifs enriched in 4–6.5 h Gro binding sites. **c** Motifs enriched in 6.5–9 h Gro binding sites

Using a de novo motif discovery algorithm (DREME), we searched the Gro binding sites at each stage for enriched motifs and then compared the motifs to the binding sites for known Drosophila transcription factors (Fig. 3). Time point 1 (1.5–4 h) Gro binding regions (Fig. 3a) are significantly enriched for Zelda (Zld) binding motifs, likely reflecting the role of Zld as a pioneer factor that may be involved in initial chromatin opening events that poise genes for subsequent expression [46]. In addition, time point 1 Gro binding regions are enriched for binding motifs for the transcription factor Dorsal, which acts as both a Gro-dependent repressor and a Gro-independent activator [2, 8, 47], the hox protein Abdominal-B (Abd-B), and the hox protein co-factor Extradenticle (Exd) [48, 49]. Time point 2 (4–6.5 h) and time point 3 (6.5–9 h) Gro binding sites (Fig. 3b, c) are enriched for binding motifs of known Gro-interacting proteins (Ventral nervous system defective [Vnd] and Sloppy paired [Slp]) [50, 51], as well as factors involved in development of the gut (dGATAe, Serpent [Srp], Forkhead [Fkh]) [52–54], cardiac development (Tinman [Tin]) [55], and anteroposterior patterning (Buttonhead [Btd], Cubitus interruptus [Ci]) [56, 57].

In the early embryo, delineation of the dorsoventral axis requires a maternally-defined concentration gradient of nuclear Dorsal along this axis [58–60]. In ventral and ventrolateral regions, Dorsal directs the repression of genes encoding dorsal ectodermal determinants, including *zerknullt* (*zen*) and *decapentaplegic* (*dpp*) by binding to ventral repression regions (VRRs) in these genes and recruiting Gro [2, 8]. In addition to repressing genes encoding dorsal ectodermal determinants, Dorsal activates mesodermal genes such as *snail* (*sna*) in the ventral region and ventral neuroectodermal genes such as *rhomboid* (*rho*) in the ventrolateral region by binding to ventral activation regions (VARs) in these genes [2, 8]. The conversion of Dorsal from an activator to a repressor results from the presence in the VRRs (but not the VARs) of binding sites for additional factors adjacent to the Dorsal binding sites [47, 61, 62]. Although it is not clear how these additional factors convert Dorsal to a repressor, it has been proposed that they assist Dorsal, which has an inherently low affinity for Gro, in Gro recruitment. As a way of assessing the simple model that Gro recruitment by Dorsal is necessary and sufficient for repression, we examined the binding of Gro to both repression and activation targets of Dorsal.

**Fig. 4:**
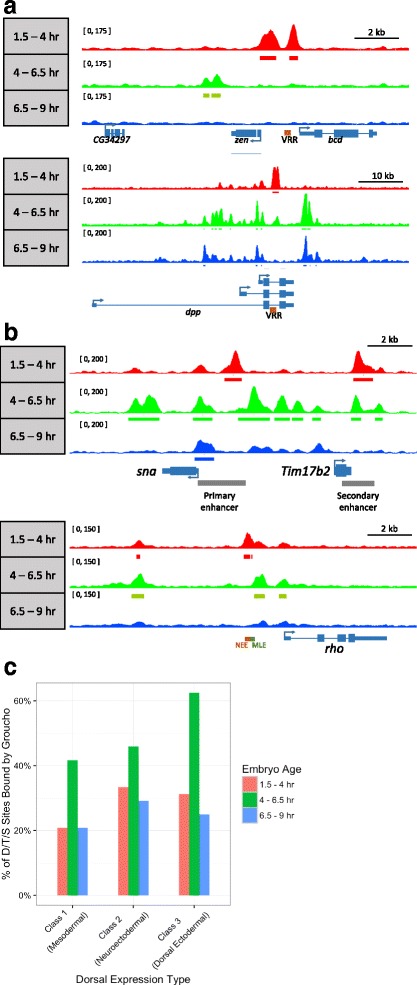
Groucho is recruited to both VRRs and VARs. **a** Genome browser views showing Gro ChIP-seq signal in genomic regions containing the two Dorsal repression targets *zen* and *dpp*. The positions of Dorsal binding site-containing VRRs in each gene are indicated. **b** Genome browser views showing Gro ChIP-seq signal in genomic regions containing the two Dorsal activation targets *sna* and *rho*. Dorsal binds and activates *sna* through a primary enhancer and a secondary (shadow) enhancer [65, 66]. Dorsal binds and activates *rho* through its neuroectodermal enhancer (nee). **c** Dorsal binds with high frequency to all three classes of Dorsal binding sites. Sites are categorized as described previously [69]. See text for a discussion of the roles of the three types of sites

As expected, we observe Gro peaks overlapping the known VRRs in the 5’ flanking region of *zen* [63] and an intron of *dpp* [64] (Fig. 4a). These VRR peaks are primarily observed in early embryos (time point 1), when Dorsal is establishing the DV axis. In both cases, we also observe Gro peaks overlapping the transcriptional start sites suggesting that Gro bound to the VRR may recruit Gro to the core promoter via a looping mechanism.

We also examined VARs in the Dorsal activation targets *rho* and *sna*. Surprisingly, Gro binds the VARs of both genes in early embryos. We observe extensive Gro binding to both the primary and “shadow” VARs in *snail*, and weaker binding to the *rho* neuroectodermal enhancer (nee), a VAR in the 5’ flanking region of *rho* [65–67] (Fig 4b). Thus, Gro recruitment may not be the critical step in converting Dorsal from an activator to a repressor.

To explore this further, we looked more broadly at localization of Gro to Dorsal binding sites. These sites can be subdivided into three classes depending on the resulting expression pattern of the regulated gene [68, 69]. Class I sites, which are low affinity sites, result in gene expression in the most ventral regions of the embryo (presumptive mesoderm), where Dorsal concentrations are highest. Class II sites are generally of higher affinity than class I sites and enable Dorsal to activate transcription at lower concentrations. As a result, these sites are active in ventrolateral regions (neuroectoderm), an area with intermediate levels of nuclear Dorsal. Class III sites are associated with genes that are repressed by Dorsal and whose expression is thereby restricted to the dorsal ectoderm where there is little or no Dorsal. In accord with what we observed at *sna* and *rho* VARs, Gro is not restricted to the class III sites but is found at all three types of sites (Fig. 4c). No single class of Dorsal site is significantly enriched over the others, indicating that Gro binds to Dorsal more frequently than previously believed, even at sites where Dorsal is activating transcription.

### Identification of high-confidence Gro targets

To incorporate our picture of Gro binding into a framework of Gro-mediated repression, we analyzed the transcriptomes of staged embryos expressing multiple dosages of Gro. These included two independent fly lines maternally overexpressing Gro. A previous study showed that these lines contain about 2–4-fold excess Gro protein relative to wild-type [23]. Additionally, we analyzed the transcriptome of embryos lacking maternally contributed functional Gro. These embryos are derived from maternal germline clones homozygous for *gro* ^*MB36*^, a lethal allele that introduces an ectopic splice site near the 5’ end of *gro.* This allele produces no detectable Gro and results in severely decreased levels of transcript, presumably due to nonsense-mediated mRNA decay [24]. Analysis of Gro transcript levels across samples at each time point confirms that overexpressing lines accumulate increased transcript levels, with the effect being greatest (about 3-fold) at time point 1 (1.5–4 h) (Additional file 4: Figure S2A). This excess transcript is partially cleared from the embryo by later time points, but does not fully return to wild-type levels over the period analyzed. Gro loss-of-function embryos failed to accumulate Gro transcripts to any significant degree across all time points indicating that the maternal contribution is dominant during this timeframe. Wild-type embryos exhibit the expected pattern of initially high levels of maternally-deposited transcript, which are gradually reduced as development proceeds.

Perturbation of Gro levels results in the misregulation of a significant proportion of the Drosophila genome at each time point (Additional file 4: Figure S2B). The Gro loss-of-function phenotype was more severe than that obtained from overexpression – over 10% of expressed genes exhibited significant changes in expression level at each time point, with the greatest effect seen in the second, 4 to 6.5 h window, compared to 2 to 5% of genes in Gro overexpression embryos.

As Gro is known to restrict the expression patterns of many developmental regulators including transcription factors, splicing factors, and signaling molecules (e.g., *tailless*, *huckebein*, *zen*, *Sxl*, *dpp*, etc.) [1, 2], many of the potential Gro targets identified through RNA-seq differential expression analysis are likely to be secondary targets that are not regulated directly by Gro. To focus on primary targets, we refined the list of potential Gro targets by integrating the RNA-seq differential expression data with Gro ChIP-seq data. This involved the use of a scoring algorithm to quantify the predictive power of Gro binding on changes in expression. A similar procedure has been successfully utilized to predict the targets of CBP, a coactivator that cooperates with Dorsal to activate gene expression in the early embryo [70], and similar methodologies have been utilized to integrate transcription factor binding and expression data in other contexts [71]. As Gro is known to be a long-range co-repressor, we modified the method to allow for greater contribution of more distant binding sites to a gene’s score (see Methods). On a per-gene basis, a “Gro occupancy score” was calculated taking into account the number, size, and positioning of any Gro peaks. Operating under a progressively relaxing score cutoff, the number of genes captured with scores above the cutoff that are up- or down-regulated upon Gro level perturbation were counted. The inflection point of the resulting response curves can then be used as an empirically-derived threshold for classifying Gro target genes.

**Fig. 5:**
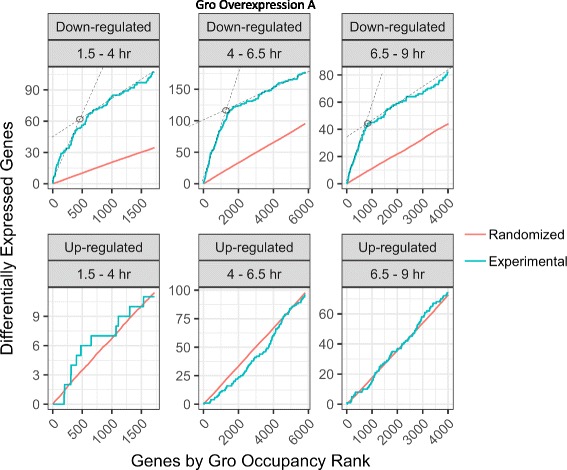
Integrating binding data (ChIP-seq) with expression data (RNA-seq) to identify high confidence Gro targets. A score corresponding to the extent of Gro occupancy within genes and adjacent areas was calculated for each gene using a previously published algorithm [35]. The algorithm was adjusted to allow for increased score contribution from regions binding more distantly from the target gene (see Methods). Plotted for each time point are the number of genes either down-regulated (top) or up-regulated (bottom) upon overexpression of Gro (vertical axis) out of the total number of genes meeting a score cutoff of decreasing stringency (horizontal axis). As the threshold Gro occupancy score decreases from left to right the number of genes that exceed this threshold (indicated by the horizontal axis labels) increases from left to right. Where a change in slope is clearly evident, the score cutoff selected for the high-confidence set of Groucho targets is indicated (circles). These are the data obtained using Gro overexpression line A. The “Experimental” curves show the data obtained using the experimentally determined up and down-regulated genes. The “Randomized” curves were obtained by generating random gene sets of the same size as the gene set used for the corresponding “Experimental” curve. Each “Randomized” curve is the average of the results obtained with 100 such random gene sets

We find that the changes in gene expression resulting from Gro overexpression (Fig. 5 and Additional file 5: S3A) are significantly more predictive of direct regulation than changes resulting from loss of Gro activity (Additional file 5: Figure S3B). In the loss-of-function experiments, the experimental curves generated using up and down-regulated genes from the RNA-seq analysis were not clearly differentiated from the control curves generated using identically sized random gene sets. This is in contrast to what we observe for the curves generated using the down-regulated genes from the overexpression experiments (see below). This may be because complete loss of maternal Gro results in such a severe developmental perturbation that indirect effects dominate. Therefore, the following analysis focuses on the overexpression data.

While the Gro/TLE family of proteins have traditionally been thought of as obligate corepressors, TLE3, a human Gro family protein, has been shown to serve primarily to activate transcription, although the mechanism remains unknown [5]. However, the differences in the distributions of up- and down-regulated genes upon overexpression (Fig. 5 and Additional file 5: S3A) can be taken as evidence against Drosophila Gro behaving as an activator. In both overexpression lines, many fewer genes with high Gro-occupancy scores were up-regulated than down-regulated. Additionally, while the down-regulated gene response curves showed clear inflection points, the up-regulated gene response curves did not. Finally, the experimental curves generated using up-regulated genes from the RNA-seq analysis were very similar to the control curves generated using identically sized random sets of genes. This stands in contrast to the curves observed for the down-regulated genes, which were clearly differentiated from the control curves. Although we cannot rule out the possibility that Gro can serve as an activator under limited and thus far undetected circumstances, we take these observations as evidence against a widespread role of Gro in transcriptional activation.

**Fig. 6:**
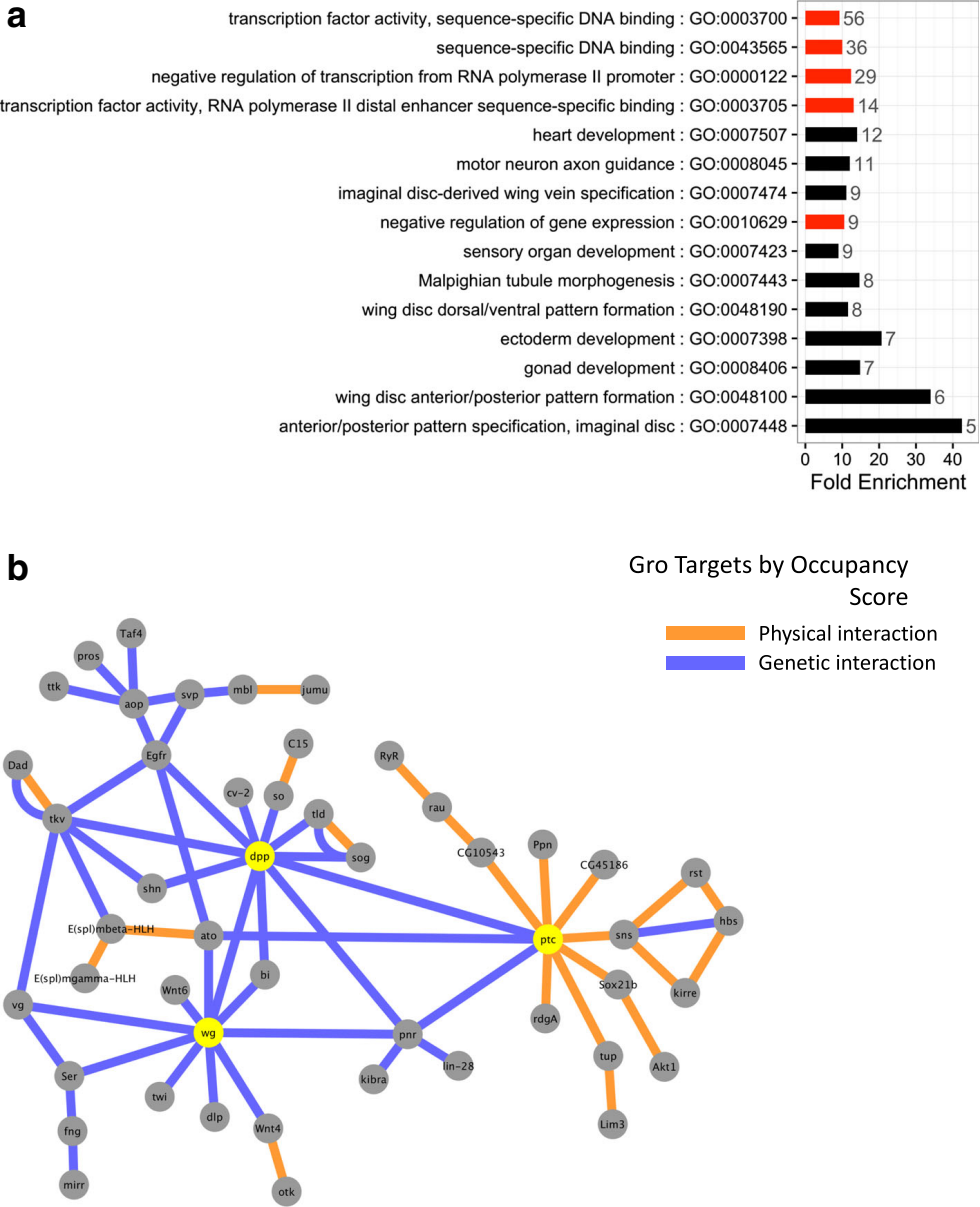
Gro target genes form a highly-interconnected network with multiple hubs. Genes that are differentially significantly down-regulated upon Gro overexpression and that exceed the ChIP-seq score cutoffs indicated by the circles in Fig. 5 define a set of 187 high-confidence targets (the full list is provided in Additional file 6: Table S3A). **a** The most significantly enriched (FDR < 0.01) gene ontology groups of high-confidence Gro target genes are uniformly related to transcriptional (red bars) and developmental (black bars) regulation, confirming the role of Gro as a high-level regulatory node in the establishment of tissue fate during development. The plot indicates fold-enrichment of the indicated groups relative to random. The numbers next to the bars indicate the numbers of genes that fall into the indicated groups, and the bars are ordered according to this number with the group containing the most genes at the top. **b** Potential Groucho-target genes were integrated into a network analysis to visualize genetic and physical interactions of these target genes. Genetic (blue edges) and physical (orange edges) interactions were obtained from a curated set maintained by FlyMine [72]. The target gene set results in highly-connected networks with multiple hubs (8 or more edges, yellow nodes) interconnected by multiple genetic interactions

Through this scoring methodology, we identified 187 potential Gro target genes (which we term “high confidence Gro targets”) across all timepoints (Additional file 6: Table S3A). Gene ontology analysis (Additional file 6: Table S3B, Figure 6a) shows that all the significantly enriched gene ontology categories (false-discovery rate < 0.01) relate to either transcriptional control (Fig. 6a, red bars) or developmental regulation (black bars). The four most frequently represented enriched gene ontology categories all relate to transcriptional control and account for 67 of the 187 high confidence targets (note that 67 is less than the total of the number of genes in the four groups as shown in Fig. 6a due to significant overlap between the gene ontology groups).

To identify potentially undocumented processes and regulatory networks in which Gro could be involved, we annotated this set of potential target genes with genetic and physical interactions curated by FlyMine [72] and integrated these results into a network to search for overrepresented groups of co-regulated genes (Fig. 6b). The resulting network features several interconnected hubs many of which correspond to components of signaling pathways, some with known Gro involvement, as well as the effectors of these pathways (see Discussion for details).

### Gro-regulated genes are enriched for promoter proximal chromatin associated RNA

**Fig. 7:**
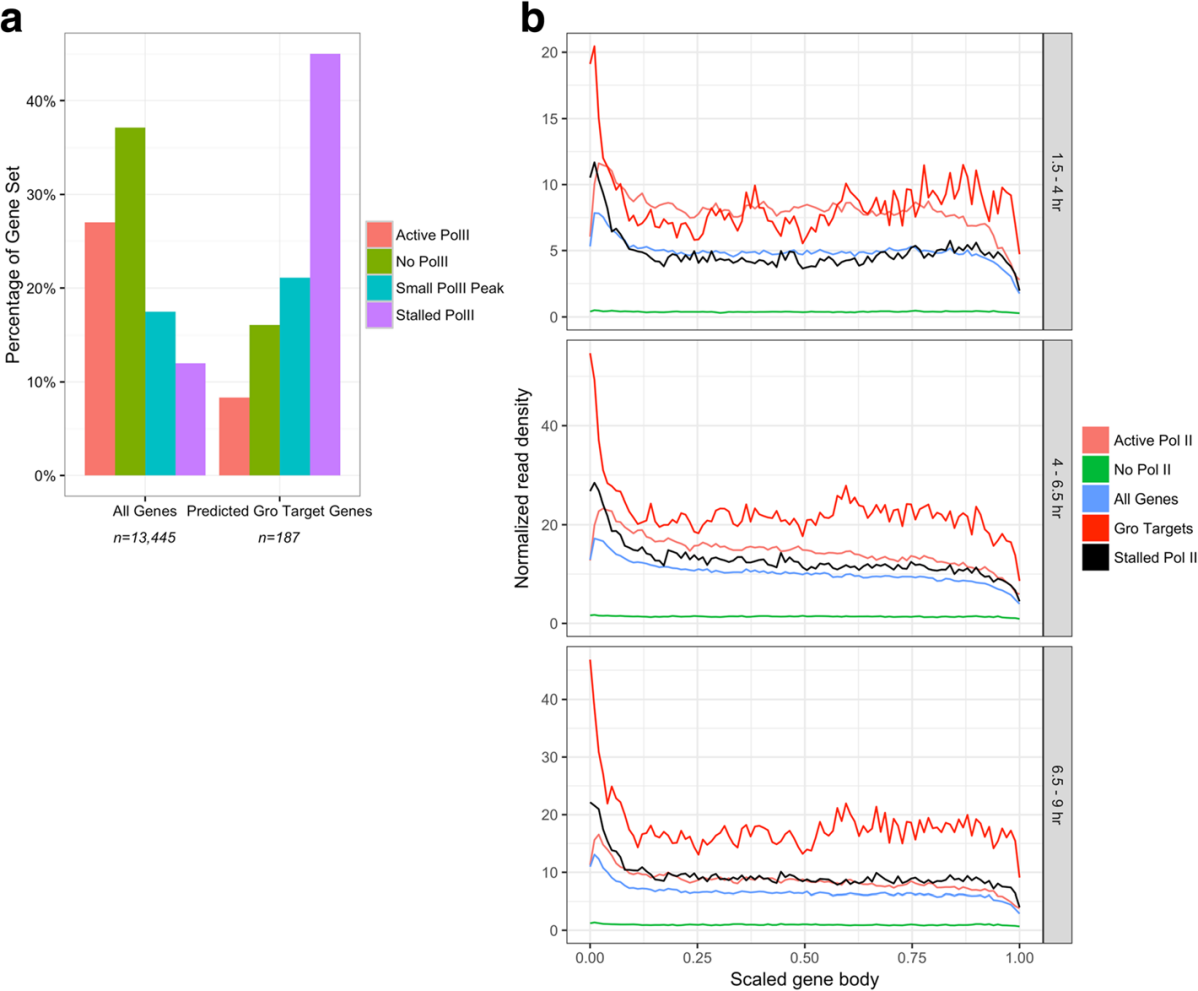
Gro regulated genes are enriched for stalled Pol II. Published data classifying all Drosophila genes into four categories of Pol II enrichment or depletion in 2–4 h embryos were used to classify all Groucho-regulated genes at each timepoint [77]. **a** Predicted Gro-regulated genes are enriched for genes classified as possessing stalled Pol II and depleted for genes possessing actively elongating Pol II. **b** Chromatin-associated transcript density across all expressed genes was calculated independently for different sets of genes at three time points. At each time window, genes predicted to be Gro regulatory targets are enriched for 5’ proximal transcript density, suggesting that these genes are enriched for stalled Pol II

Promoter-proximal pausing of Pol II has been identified as a crucial step in gene regulation. Pausing was originally characterized in *Drosophila* at heat-shock genes, where it was thought to facilitate rapid induction of gene expression upon receipt of an appropriate regulatory signal [73]. Since this discovery, polymerase stalling has been found to be a ubiquitous regulatory mechanism in both Drosophila and humans [74–77]. When we analyzed published Pol II ChIP-chip data [77], we found that the 187 high confidence Gro targets identified as described in the previous section are significantly enriched for genes containing stalled Pol II, suggesting a possible connection between Gro-mediated repression and Pol II pausing (Fig. 7a). As an alternative measure of Pol II pausing, we analyzed published global run-on-sequencing (Gro-seq) data. This analysis (Additional file 7: Figure S5) shows once again that the high confidence targets are significantly enriched for genes with high pausing indices (those in the top quartile) relative to genes with low pausing indices (those in the bottom three quartiles).

To further explore this association, we isolated chromatin-associated RNA from embryos at multiple stages of development (Additional file 8: Figure S4A, B), depleted it of rRNA (Additional file 8: Figure S4C), and subjected it to high-throughput sequencing. The “nascentness” of the fractionated pre-mRNA was validated through comparison to total mRNA libraries. Chromatin-associated mRNA was found to have increased levels of intron retention (Additional file 9: Table S4), a strong 5’ bias in read distribution (Fig. 7b), and was enriched for transcripts synthesized in early embryogenesis, in contrast to the large number of maternally-synthesized transcripts found in mature early-embryonic RNA (Additional file 10: Table S5).

The chromatin-associated RNA-seq data provide information about the distribution of nascent transcript lengths arising from each gene, which serves as an indicator of the patterns of Pol II positioning within genes. Comparing the 187 high confidence Gro targets with all genes, genes containing active Pol II, genes containing stalled Pol II, and genes containing no Pol II, we see that, at all three timepoints, the Gro targets exhibit a significantly greater promoter-proximal to gene body chromatin-associated RNA ratio than does the genome as a whole (Fig. 7b). This is consistent with the conclusion that Gro-repressed genes are enriched for paused Pol II.

## Discussion

### Gro binds dynamically to discrete sites in chromatin

We have identified thousands of novel Gro-recruitment sites throughout the Drosophila genome. The majority (almost 60%) of these sites are only occupied during one of the three time windows we sampled during the first nine hours of embryogenesis and less than 10% are constitutively occupied throughout this period. This dynamic pattern of Gro occupancy likely reflects the shifting availability of sequence-specific transcription factors able to recruit Gro to chromatin.

Gro recruits histone deacetylase HDAC1/Rpd3, leading to localized deacetylation of histones and a consequent increase in nucleosome density and repression [15–17]. Deacetylation of histone H3 and H4 tails is also observed at a distance from the site of Gro recruitment, suggesting that long-range repression by Gro may involve spreading of Gro from the site of recruitment [78, 79]. This hypothesis is consistent with the ability of Gro to bind hypoacetylated histones and with a role for Gro oligomerization in repression [9–11, 19, 20]. However, data presented here looking at temporally staged embryos and elsewhere looking at tissue culture cells [22] show that Gro binds to discrete sites and not to continuous stretches of chromatin. The majority of Gro binding occurs in clusters of multiple localized peaks less than 1 kb in width. This is inconsistent with the idea that spreading is a one-dimensional process in which Gro polymerizes along the chromatin fiber. We thus propose that Gro oligomers serve to transfer Gro and associated histone marks to sites distant from Gro recruitment via a looping mechanism, a proposal that is consistent with our finding that Gro peaks tend to cluster and that Gro is often associated with transcriptional start sites. As Gro tetramers can crosslink chromatin arrays in vitro [43], the presence of these peak clusters may represent the extension of this function to in vivo contexts. Mutations to the Q-domain which disrupt self-association result in misregulation of a subset of Gro targets [22]. This differential requirement for oligomerization can be explained by our observation that Gro frequently localizes within genes and near transcription start sites in the embryo, where the dependence on efficient oligomerization-mediated transfer of histone marks would be reduced in comparison with recruitment to distant silencing regions.

Our finding that *sog* is likely to be a direct Gro target suggests an alternative to the idea that Gro bound to distal sites serves to transfer Gro to promoter proximal regions and that this leads to repression. Chromatin confirmation capture assays suggest that activation of *sog* may require loop formation between distal regulatory elements and the proximal promoter regions and that repression of *sog* may involve an anti-looping mechanism [80]. Thus, it is possible that Gro is recruited to promoter proximal regions directly by promoter proximal-bound sequence specific transcription factors where it serves to inhibit loop formation.

### Gro recruitment is not sufficient for repression

Our data show that Groucho binds to many genes that it does not appear to repress. While this could reflect a lack of adequate sensitivity in the expression profiling analysis, we favor the idea that Gro-recruitment is not sufficient for repression. This is suggested by our analysis of Dorsal regulatory targets.

We find that Gro is recruited to the Dorsal-binding VRRs of *zen* and *dpp*, two well-characterized ventrally-repressed genes, consistent with Dorsal-mediated recruitment and repression. Gro occupies the VRRs in these genes, as well as their transcription start sites consistent with the idea that Gro may be delivered from the VRR to the core promoter region, perhaps by a mechanism involving DNA looping.

The Dorsal repression targets *dpp* and *tld* are among our high confidence Gro targets. This indicates that overexpression of Gro results in reduced *dpp* and *tld* expression, an effect that is likely direct. This reduced expression supports the idea that the threshold Dorsal concentration required for repression is sensitive to Gro concentration [1, 23]. Thus in the Gro overexpressing embryos, the domain of Dorsal-mediated repression may extend into the dorsal ectodermal region where the levels of nuclear Dorsal are usually too low to support repression.

Unlike many Gro-dependent repressors (e.g., the Hairy-Enhancer of Split [HES] family repressors), Dorsal is not a dedicated repressor, but also activates expression of certain targets [81]. Surprisingly, we find that Gro is recruited to Dorsal binding VARs in these Dorsal activation targets. This was unexpected as it was believed that Dorsal by itself was insufficient for Gro recruitment due to its intrinsically low affinity for Gro. Rather, it was thought that Dorsal plus additional factors bound to sites adjacent to the Dorsal binding sites in VRRs were required to form a high-affinity platform for Gro recruitment [8, 62]. We thus suggest that something about the particular nature of the interaction between a sequence-specific factor and Gro, rather than Gro recruitment per se, may be critical in determining whether or not repression will occur. For example, it is possible that the kinetic stability of a repression complex (a “repressosome”) including multiple sequence specific factors and multiple coregulators is a critical determinant of whether or not repression will occur. This idea is consistent with experiments in which fusion to Dorsal of the WRPW high affinity Gro binding motif found in HES family repressors was found to convert Dorsal into a dedicated repressor [82]. This idea is also consistent with our finding that the Dorsal activation targets *twi* and *sog* are among our high confidence Gro repression targets, since sufficiently high Gro concentration may stabilize an otherwise unstable co-repressor complex. An alternative possibility that we cannot rule out is that Gro is recruited to VARs by factors other than Dorsal and that this serves to help ensure complete repression of Dorsal activation targets in tissues where they are not normally expressed.

### High confidence Gro targets define a highly interconnected gene regulatory network

Perturbation of Gro activity has severe consequences on the embryonic developmental program [3]. We observe hundreds of misregulated genes at each developmental stage, confirming that Gro is thoroughly integrated into the gene regulatory network. This network is highly sensitive to increased Gro dosage, indicating that endogenous Gro is not expressed at levels that result in saturated interaction with its targets, a finding that is consistent with the idea that Gro is the target of extensive post-translational regulation [1, 83]. These potential Gro targets were filtered using combinations of RNA-seq and ChIP-seq data to obtain lists enriched for high confidence direct targets of Gro repression. This list contains 187 genes regulated by Gro at one or more stage in the embryo. Direct Gro targets are enriched for transcription factors controlling multiple aspects of gene expression, explaining how altering Gro levels can generate widespread changes in gene expression.

We note that the approach that we used to identify high confidence targets assumes that the influence of a Gro peak on the expression of a gene tends to decrease as the gene gets further away from the Gro peak. This is a somewhat controversial assumption. For example, a recent analysis showed that long-range looping interactions involving enhancers are very common, with the majority of such interactions occurring at distances of >50 kb [84]. However, the fact that plots of Gro occupancy threshold vs. number of repressed genes yield curves that are very different from those obtained from random sets of genes tends to validate our approach for identifying high confidence targets.

The high confidence Gro targets identified here show that Gro regulates both upstream and downstream elements of a highly-interconnected network of signaling pathways. These include multiple signaling pathways with known Gro involvement, including the Dpp, Wingless, and Epiderimal Growth Factor Receptor (EGFR) signaling pathways [1, 83]. Furthermore, we also detect novel involvement with downstream effectors of these pathways, such as *pannier* (*pnr*), *atonal* (*ato*), and *patched* (*ptc*). The presence in the network of a hub around *pnr*, which encodes a transcription factor involved in cardiogenesis [85], is of interest because it suggests an undocumented contribution of Gro to the regulation of cardiac development. This connection is consistent with our finding that Gro binding sites are enriched for Tin binding motifs, since Tin works together with Pnr to regulate cardiac gene expression [86–88]. Finally, the network captures multiple Dorsal repression targets such as *dpp* [64], *tolloid* (*tld*) [89] and *short gastrulation* (*sog*) [90, 91], consistent with the fact that Dorsal represses via Gro [47].

### Gro repression targets are enriched for promoter proximal Pol II

We find that these high confidence direct targets of Gro-mediated repression are enriched for paused Pol II in the embryo. Consistent with this conclusion, chromatin associated RNA-seq shows that chromatin associated RNA is significantly enriched for transcription start site proximal RNA. These findings suggest that Gro-mediated repression may involve promoter proximal pausing of Pol II.

The manner in which Pol II pausing is utilized to regulate transcription remains poorly understood, although multiple non-exclusive mechanisms have been proposed, [92]. One of these mechanisms posits that sustained or transient pausing facilitates the participation of additional regulatory elements in the determination of transcriptional activity [93]. This allows the expression level of a gene to be regulated through multiple, independent pathways, potentially leading to synergistic effects on rates of transcription [94]. Combinatorial control of gene expression is a common regulatory motif in eukaryotes, and thus mechanisms that influence expression both before the assembly of the Pol II complex and after transcriptional initiation could be used to integrate multiple regulatory inputs.

Given that genes possessing stalled Pol II often continue to be expressed at high levels [93, 95], Pol II stalling in Gro-regulated genes may not be a primary mechanism of repression, but instead could indicate that these genes are primed for rapid activation once Gro-mediated repression is relieved. The ability of this repression to be rapidly reversed may be an important aspect of Gro function during development.

## Conclusions

To advance our understanding of the mechanisms of development, we have analyzed the patterns of Gro recruitment throughout the Drosophila embryonic genome. We carried out this analysis on Drosophila embryos at multiple stages in the first nine hours of embryogenesis. This is a period when the gene expression profile is rapidly evolving, and we find that the binding of Gro is likewise rapidly changing during this period. The binding sites average three to four nucleosomes in length, and tend to cluster both in distal regions and near the transcriptional start sites of genes. This pattern of binding is inconsistent with one-dimensional spreading of Gro along chromatin and is more supportive of a mode of spreading between cis-regulatory modules and promoters that involves DNA looping. In the future, it may be possible to further test this model using chromatin conformation capture assays in embryos with altered levels of Gro to ascertain the role of Gro in the formation of chromatin loops.

Our data also indicate that Gro binding is not the determinative step in Gro-mediated repression as Gro is recruited to both activation and repression targets of the transcription factor Dorsal. We thus suggest that some property of the Gro-containing repressosome (e.g., its kinetic stability) is what determines whether or not repression occurs.

We have combined our analysis of Gro binding with measurements of changes in gene expression upon perturbation of Gro levels to identify a set of high confidence targets of Gro repression. Many of these targets comprise a highly interconnected network of genes involved in transcriptional regulation and multiple signaling pathways including the Dpp, Wingless, and EGFR pathways. Analysis of chromatin-associated RNA levels at these target genes reveals that they are enriched for promoter proximal transcripts consistent with a role for Pol II pausing in Gro-mediated repression. Future tests of this model could involve looking at the effect of Gro loss-of-function or overexpression on the extent of Pol II pausing at these high confidence targets using such approaches as Pol II ChIP or GRO-seq.

## Additional files

Additional file 1: Table S1. Sizes and mapping characteristics of sequencing libraries generated for ChIP-seq, poly(A) + RNA-seq, and chromatin-associated RNA-seq analyses. (DOCX 158 kb)
Additional file 2: Table S2. Oligonucleotides used for rRNA depletion. (DOCX 78 kb)
Additional file 3: Figure S1. Antibody validation (A) Chromatin isolated and sheared exactly as for the ChIP-seq analysis was subjected to immunoprecipitation with the indicated amounts (in μl) of affinity purified antibody against the Gro GP domain used for the ChIP-seq analysis, and then probed in a western blot with both an anti-Gro monoclonal antibody (mAb) or the anti-GP antibody. The band indicated by the asterisk is a cross-reacting protein that is recognized in the western blot but that is not efficiently immunoprecipitated by the anti-GP antibody. Ab HC – antibody heavy chain. (B) Heat map showing overlap (Jacard similarity coefficient [96]) between the peaks called in the duplicate ChIP-seq experiments at each time point. (C) Representative genome browser tracts comparing duplicate ChIP-seq experiments. (D and E) Comparison of Gro binding patterns obtained by ChIP-seq using our anti-GP antibody with that obtained by ChIP-chip (0–12 hr embryos; modENCODE #597) and ChIP-seq (white pre-pupae; modENCODE #4981) using independently derived antibodies [40]. (PDF 588 kb)
Additional file 4: Figure S2. Fractions of genes showing altered expression in Gro overexpression and Gro LOF embryos. (A) Normalized Gro transcript expression levels were calculated at each timepoint. (B) Maternal Gro deficiency results in a large proportion (>10%) of expressed genes becoming misregulated in the Drosophila embryo across all time points. Overexpression of Gro results in a smaller but still significant alteration of the embryonic transcription profile. (PDF 451 kb)
Additional file 5: Figure S3. Numbers of differentially expressed genes in Gro overexpression and loss-of-function embryos as a function of Gro occupancy score. These graphs are similar to those in Fig. 5, except that they incorporate the RNA-seq data from (A) Gro overexpression line B, and (B) the Gro maternal loss-of-function embryos. For (A) each “Randomized” curve is the average (linear interpolation) of 100 trials with 100 sets of randomly generated genes. Randomized gene sets contained identical numbers of up- and down-regulated genes as found in the original data. For (B), rather than averaging the results from the random gene sets, the results for each random gene set are plotted as a separate curve. (PDF 2313 kb)
Additional file 6: Table S3. 187 high confidence Gro targets. The following tables are provided in an Excel spreadsheet: (A) Occupancy scores and differential expression levels and (B) Top gene ontology categories (FDR < 0.01) (XLSX 71 kb)
Additional file 7: Figure S5. Gro-regulated genes are enriched for genes with high pausing index based on GRO-seq analysis. The prevalence of Pol II promotor-proximal pausing in predicted Gro targets was calculated across all timepoints (1.5–9 hrs, A) or in the first timepoint only (1.5–4 hrs, B). An index of promotor-proximal PolII pausing was obtained from GRO-seq data generated in 2–2.5 hr old wild-type embryos (see Saunders et al., 2013 for details [97]). The resulting genes were split into quartiles based on pausing index and compared to predicted Gro targets. Approximately 41% of predicted Gro targets in 1.5–4 hr embryos were found in the upper quartile, indicating a strong association between Gro regulation and polymerase pausing. This enrichment for paused genes is highly significant, with a *p*-value of < 10^−10^ by a Wilcoxon rank sum test. (PDF 758 kb)
Additional file 8: Figure S4. Validation of enrichment of chromatin-associated RNA from total embryonic RNA. (A) Protein components of embryo fractions utilized for RNA-seq were visualized with SDS-PAGE. Isolated chromatin was enriched for multiple bands in the 15 to 19 kDa range consistent with histone core proteins. (B) Immulobloting reveals the lack of a cytoplasmic marker (tubulin) in the nucleoplasmic and chromatin fractions, as well as the presence of histone H3 in the chromatin fraction. (C) As chromatin-associated RNA is largely non-polyadenylated, poly(A) + affinity techniques commonly utilized to isolate mRNA from the much larger pools of non-coding transcripts could not be utilized. An affinity depletion protocol was instead used to remove the major *D. melanogaster* rRNA transcripts as well as other non-coding RNAs prior to high-throughput sequencing. Depletion of the two major rRNA species (28 s and 18 s) was confirmed via Agilent Bioanalyzer RNA profiles. Large rRNA peaks in the input indicate the RNA pool underwent minimal degradation during fractionation and purification. (PDF 427 kb)
Additional file 9: Table S4. Overlap of sequenced reads from chromatin-associated and total poly(A) + RNA-seq libraries. (DOCX 44 kb)
Additional file 10: Table S5. Low and High abundance transcripts from chromatin-associated RNA-seq. (DOCX 112 kb)

## Abbreviations

Abd-B: Abdominal-B
ato: Atonal
btd: Buttonhead
ChIP: Chromatin immunoprecipitation
ci: Cubitus interruptus
dCtBP: Drosophila C-terminal binding protein
dl: Dorsal
dpp: Decapentaplegic
EGFR: Epidermal growth factor receptor
exd: Extradenticle
FDR: False discovery rate
fkh: Forkhead
GO: Gene ontology
gro: Groucho
HDAC1: Histone deacetylase 1
HES: Hairy enhancer of split
mRNA: Messenger ribonucleic acid
pnr: Pannier
ptc: Patched
rho: Rhomboid
RNA Pol II: RNA Polymerase II
RNA: Ribonucleic acid
slp: Sloppy paired
sna: Snail
sog: Short gastrulation
srp: Serpent
sxl: Sex lethal
tin: Tinman
tld: Tolloid
TLE: Transducin-like enhancer of split
TSS: Transcription start site
VAR: Ventral activation region
vnd: Ventral neuroblast defective
VRR: Ventral repression region
wg: Wingless
zen: Zerknullt
zld: Zelda
