# Supplementary material for: Mechanisms of Groucho-mediated repression revealed by genome-wide analysis of Groucho binding and activity
Source: BMC Genomics. 2017 Feb 28;18:215. doi: 10.1186/s12864-017-3589-6 (PMC5331681; doi:10.1186/s12864-017-3589-6)
Supplement: Additional file 1: Table S1. — Sizes and mapping characteristics of sequencing libraries generated for ChIP-seq, poly(A) + RNA-seq, and chromatin-associated RNA-seq analyses. (DOCX 158 kb) [file 12864_2017_3589_MOESM1_ESM.docx]

**Table S1. Sizes and mapping characteristics of sequencing libraries generated for ChIP-seq, poly(A)+ RNA-seq, and chromatin-associated RNA-seq analyses**

|  | **Index Number** | **Barcode** | **Number of sequences** | **Number of uniquely mapped sequences** | **Percentage of uniquely mapped sequences** |
| --- | --- | --- | --- | --- | --- |
| **ChIP-seq libraries** |  |  |  |  |  |
| Input | 3 | AGTGAG | 26,804,524 | 19,349,084 | 72% |
| Input | 8 | GTCGTA | 22,770,618 | 16,367,817 | 72% |
| Gro ChIP 1.5 - 4.0 hr | 4 | GCACTA | 22,276,144 | 8,242,625 | 37% |
| Gro ChIP 1.5 - 4.0 hr | 5 | ACCTCA | 23,743,733 | 5,992,455 | 25% |
| Gro ChIP 4.0 - 6.5 hr | 6 | GTGCTT | 19,973,255 | 7,925,951 | 40% |
| Gro ChIP 4.0 - 6.5 hr | 7 | AAGCCT | 17,755,470 | 8,440,270 | 48% |
| Gro ChIP 6.5 - 9.0 hr | 1 | AACCAG | 18,066,141 | 7,979,752 | 44% |
| Gro ChIP 6.5 - 9.0 hr | 2 | TGGTGA | 22,367,998 | 8,179,454 | 37% |
|  |  |  |  |  |  |
| **Chromatin-associated RNA-seq libraries** | |  |  |  |  |
| Nascent 1.5 - 4.0 hr | 1 | ATCACG | 15,630,090 | 13,396,998 | 86% |
| Nascent 1.5 - 4.0 hr | 5 | ACAGTG | 17,158,942 | 14,402,702 | 84% |
| Nascent 4.0 - 6.5 hr | 4 | TGACCA | 21,322,745 | 19,102,357 | 90% |
| Nascent 4.0 - 6.5 hr | 10 | TAGCTT | 18,073,092 | 15,913,811 | 88% |
| Nascent 6.5 - 9.0 hr | 8 | ACTTGA | 18,595,553 | 15,747,237 | 85% |
| Nascent 6.5 - 9.0 hr | 11 | GGCTAC | 15,198,232 | 13,952,494 | 92% |
|  |  |  |  |  |  |
| **poly(A)+ RNA-seq libraries** |  |  |  |  |  |
| wild-type 1.5 - 4.0 hr | 1 | ATCACG | 23,204,999 | 19,329,012 | 83% |
| wild-type 1.5 - 4.0 hr | 27 | ATTCCT | 30,491,312 | 23,513,268 | 77% |
| wild-type 4.0 - 6.5 hr | 10 | TAGCTT | 32,894,298 | 27,894,616 | 85% |
| wild-type 4.0 - 6.5 hr | 21 | GTTTCG | 32,001,276 | 25,433,743 | 79% |
| wild-type 6.5 - 9.0 hr | 22 | CGTACG | 28,018,855 | 23,708,623 | 85% |
| wild-type 6.5 - 9.0 hr | 9 | GATCAG | 23,654,873 | 20,544,064 | 87% |
| Gro Overexpression A 1.5 - 4.0 hr | 3 | TTAGGC | 37,701,284 | 31,299,972 | 83% |
| Gro Overexpression A 1.5 - 4.0 hr | 25 | ACTGAT | 33,055,437 | 24,765,540 | 75% |
| Gro Overexpression A 4.0 - 6.5 hr | 11 | GGCTAC | 26,637,697 | 21,644,279 | 81% |
| Gro Overexpression A 4.0 - 6.5 hr | 20 | GTGGCC | 32,662,754 | 25,756,330 | 79% |
| Gro Overexpression A 6.5 - 9.0 hr | 23 | GAGTGG | 28,945,740 | 24,103,897 | 83% |
| Gro Overexpression A 6.5 - 9.0 hr | 8 | ACTTGA | 27,105,089 | 22,979,797 | 85% |
| Gro Overexpression B 1.5 - 4.0 hr | 8 | ACTTGA | 39,759,462 | 32,163,967 | 81% |
| Gro Overexpression B 1.5 - 4.0 hr | 23 | GAGTGG | 33,277,102 | 26,463,812 | 80% |
| Gro Overexpression B 4.0 - 6.5 hr | 20 | GTGGCC | 41,608,339 | 33,500,240 | 81% |
| Gro Overexpression B 4.0 - 6.5 hr | 11 | GGCTAC | 30,191,854 | 24,255,811 | 80% |
| Gro Overexpression B 6.5 - 9.0 hr | 25 | ACTGAT | 32,776,678 | 26,833,728 | 82% |
| Gro Overexpression B 6.5 - 9.0 hr | 3 | TTAGGC | 29,247,470 | 24,800,966 | 85% |
| Gro loss-of-function 1.5 - 4.0 hr | 2 | CGATGT | 67,665,979 | 63,734,396 | 94% |
| Gro loss-of-function 1.5 - 4.0 hr | 6 | GCCAAT | 55,094,526 | 51,906,482 | 94% |
| Gro loss-of-function 4.0 - 6.5 hr | 4 | TGACCA | 59,363,155 | 55,492,890 | 93% |
| Gro loss-of-function 4.0 - 6.5 hr | 7 | CAGATC | 42,101,460 | 39,465,785 | 94% |
| Gro loss-of-function 6.5 - 9.0 hr | 5 | ACAGTG | 46,462,702 | 43,404,870 | 93% |
| Gro loss-of-function 6.5 - 9.0 hr | 12 | CTTGTA | 42,806,093 | 39,686,467 | 93% |
