# Supplementary material for: Mechanisms of Groucho-mediated repression revealed by genome-wide analysis of Groucho binding and activity
Source: BMC Genomics. 2017 Feb 28;18:215. doi: 10.1186/s12864-017-3589-6 (PMC5331681; doi:10.1186/s12864-017-3589-6)
Supplement: Additional file 2: Table S2. — Oligonucleotides used for rRNA depletion. (DOCX 78 kb) [file 12864_2017_3589_MOESM2_ESM.docx]

**Table S2: Oligonucleotides used for rRNA depletion**

| Target | Sequence |
| --- | --- |
| 2s | CTTACAACCCTCAACCATATGTAGTCCAAGCAGC |
| 18s | CAATAATGATCCTTCCGCAGGTTC |
| 5.8s | CAGCATGGACTGCGATATGCGTTC |
| 28s alpha | ATTTTCGCTTTCGCCTTGAAC |
| 28s Beta | TCGAATCATCAAGCAAAGGATAAGC |
| 28s | GTGTTAATTAGCTATAAATAGCTAAAAAACTAATC |
| 28s | CAGGTTACGGAATTGGAACCGTATTCCCTTTCGTT |
| 28s | CAATCTTCAGAGCCAATCCTTATCCCGAAGTTACG |
| 28s | GCCCGTTCCCTTGGCTGTGGTTTCGCTAG |
| 18s | GAACAGAGGTCTTATTTCATTATCCCATGCACAGA |
| 18s | CGGTACAAGACCATACGATCTGCATGTTATCTAGA |
| 18s | TTTAATTGCATGTATTAGCTCTAGAATTACCACAG |
| 5s | AAGTTGTGGACGAGGCCAACAACACGCGGTGTTCCC |
| 5'_end_of_rRNA | TATTCCTATTATCCGCGGAG |
| 5'_end_of_rRNA | CCATTCGAATACGGCCATTT |
| nodavirus RNA1 | ACCTCCGCCCTTTCGGGCTAGAAC |
| nodavirus RNA2 | ACCTTAGTCGGCTGACTTAAACTGTC |
| totivirus SW-2009a | CGACTATATCTTCTGCGTTATCCAGC |
| oligo dT | TTTTTTTTTTTTTTT |
