# Supplementary figures and images for: Mechanisms of Groucho-mediated repression revealed by genome-wide analysis of Groucho binding and activity
Source: BMC Genomics. 2017 Feb 28;18:215. doi: 10.1186/s12864-017-3589-6 (PMC5331681; doi:10.1186/s12864-017-3589-6)

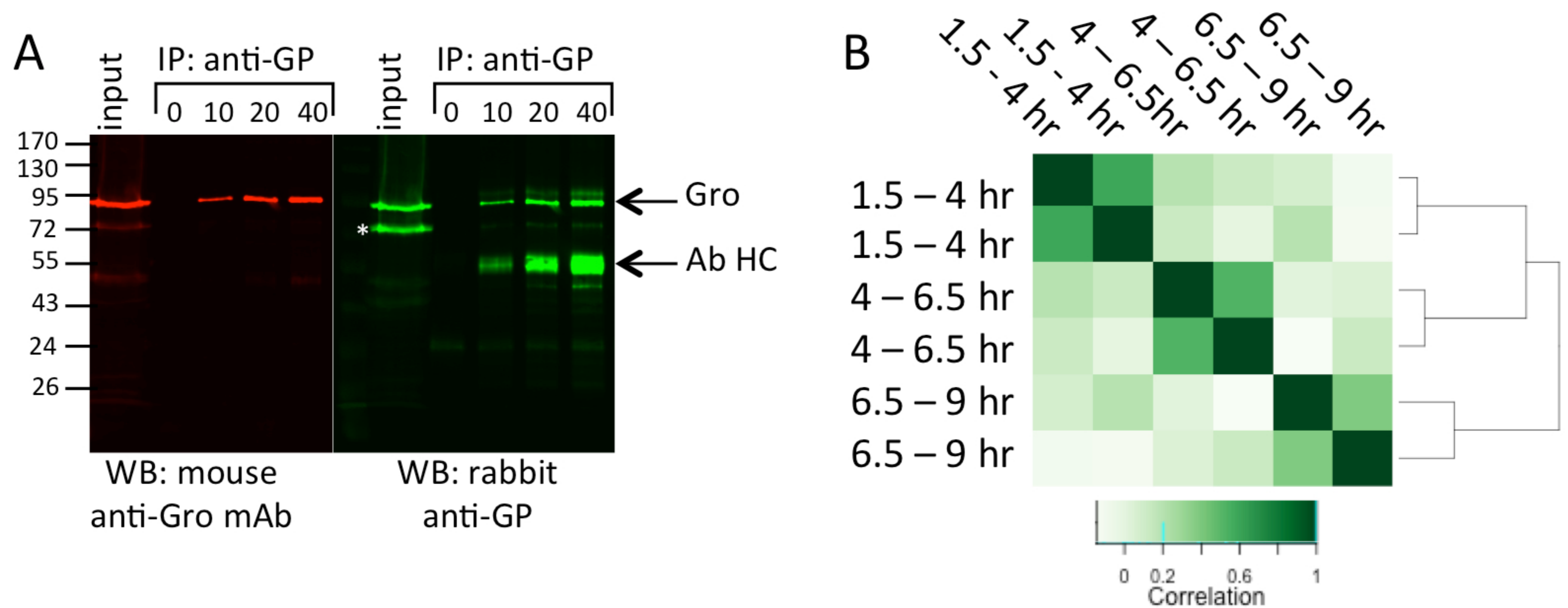

**C**

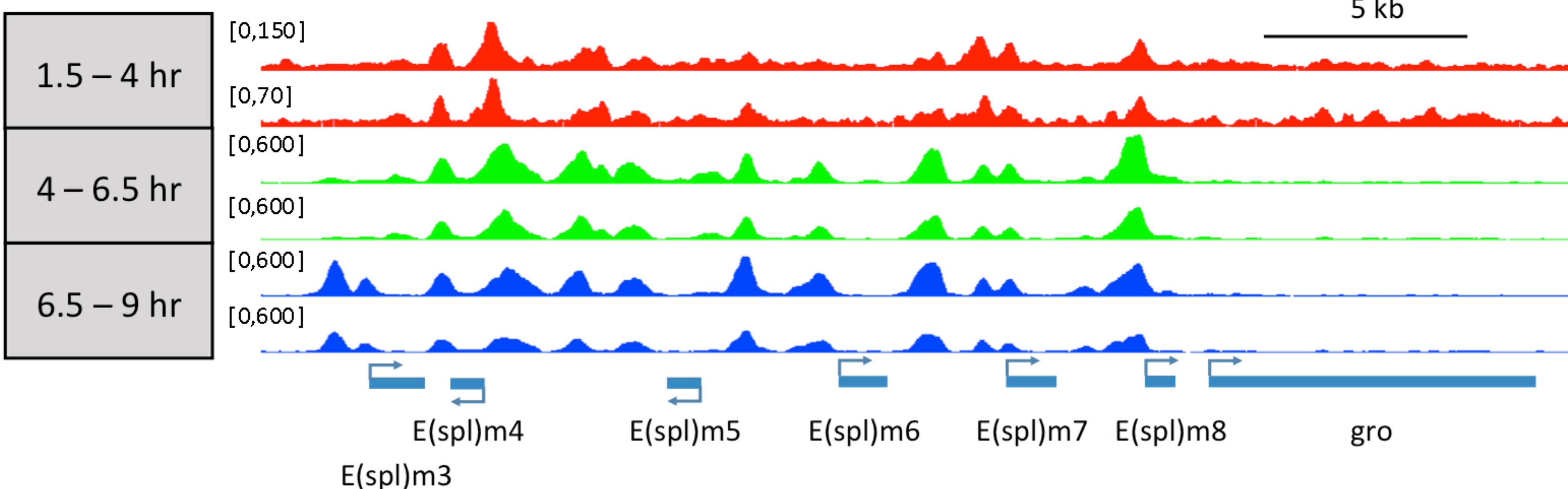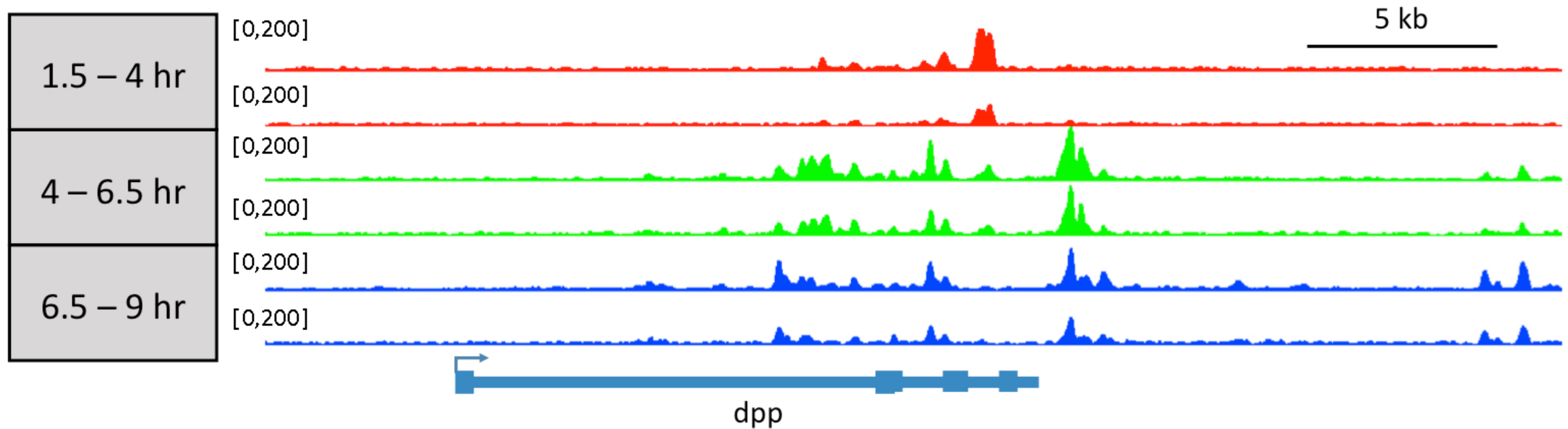

**D**

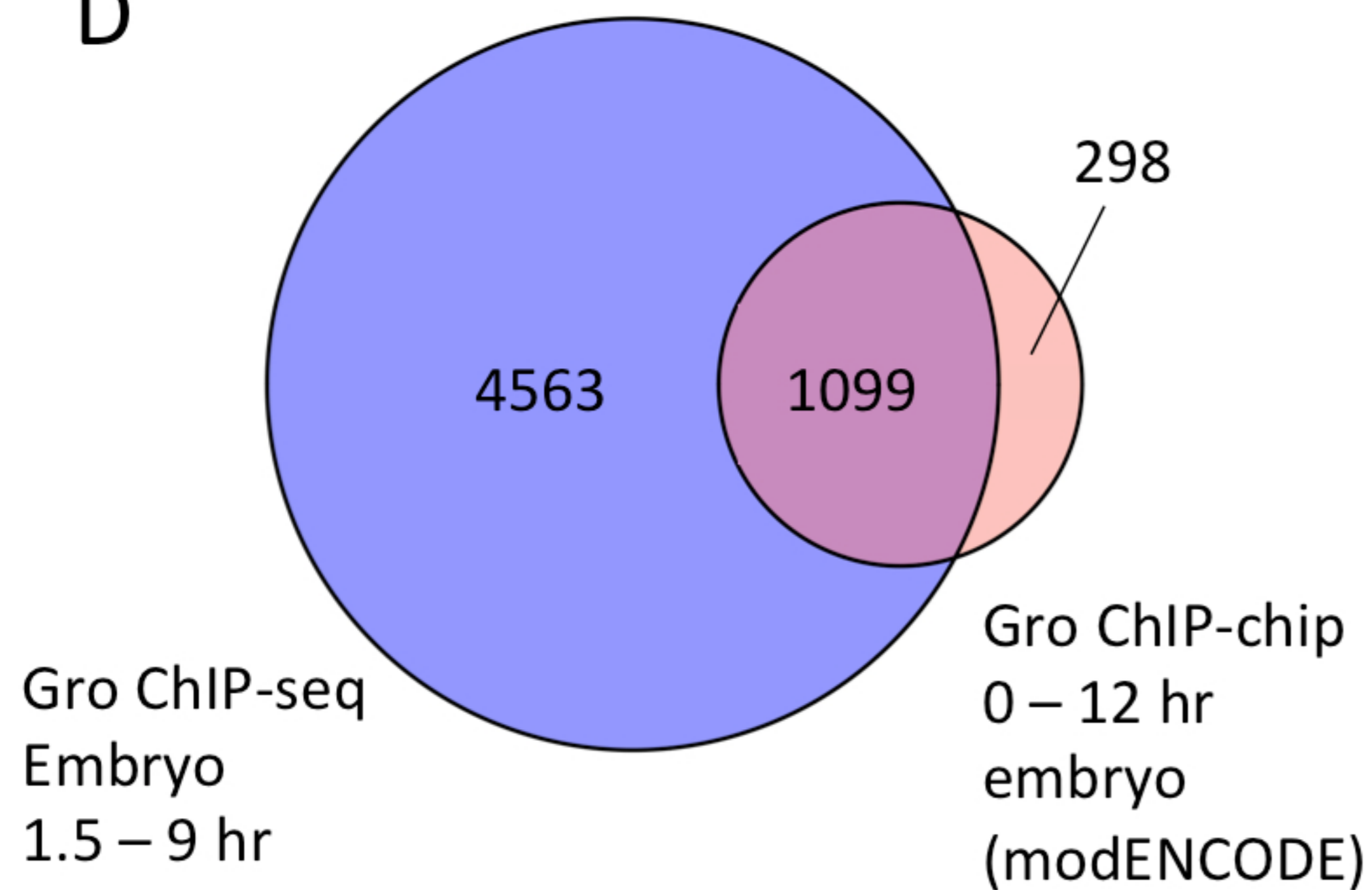

**E**

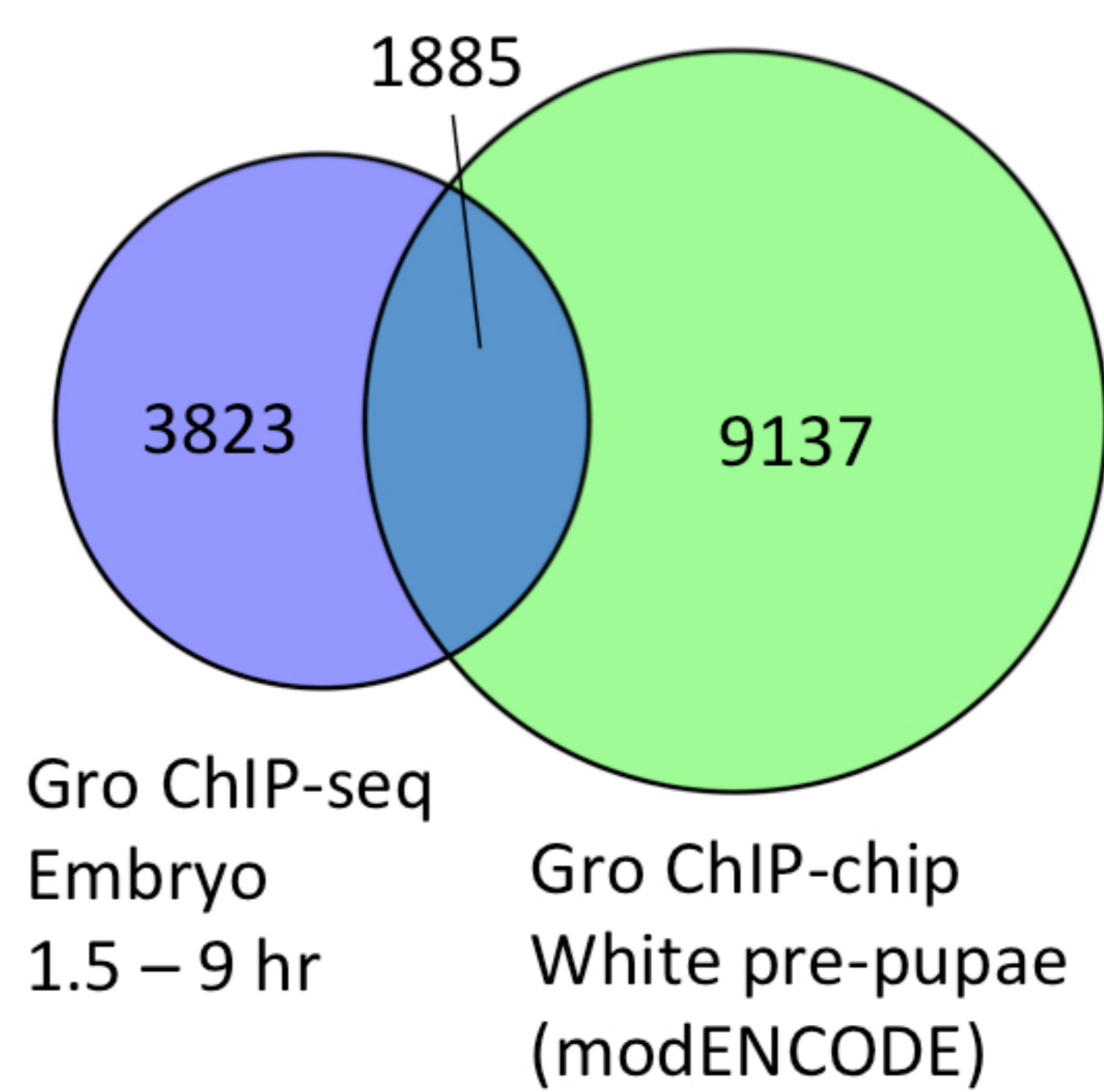

Figure S1

Supplement: Additional file 3: Figure S1. — Antibody validation (A) Chromatin isolated and sheared exactly as for the ChIP-seq analysis was subjected to immunoprecipitation with the indicated amounts (in μl) of affinity purified antibody against the Gro GP domain used for the ChIP-seq analysis, and then probed in a western blot with both an anti-Gro monoclonal antibody (mAb) or the anti-GP antibody. The band indicated by the asterisk is a cross-reacting protein that is recognized in the western blot but that is not efficiently immunoprecipitated by the anti-GP antibody. Ab HC – antibody heavy chain. (B) Heat map showing overlap (Jacard similarity coefficient [96]) between the peaks called in the duplicate ChIP-seq experiments at each time point. (C) Representative genome browser tracts comparing duplicate ChIP-seq experiments. (D and E) Comparison of Gro binding patterns obtained by ChIP-seq using our anti-GP antibody with that obtained by ChIP-chip (0–12 hr embryos; modENCODE #597) and ChIP-seq (white pre-pupae; modENCODE #4981) using independently derived antibodies [40]. (PDF 588 kb) [file 12864_2017_3589_MOESM3_ESM.pdf]

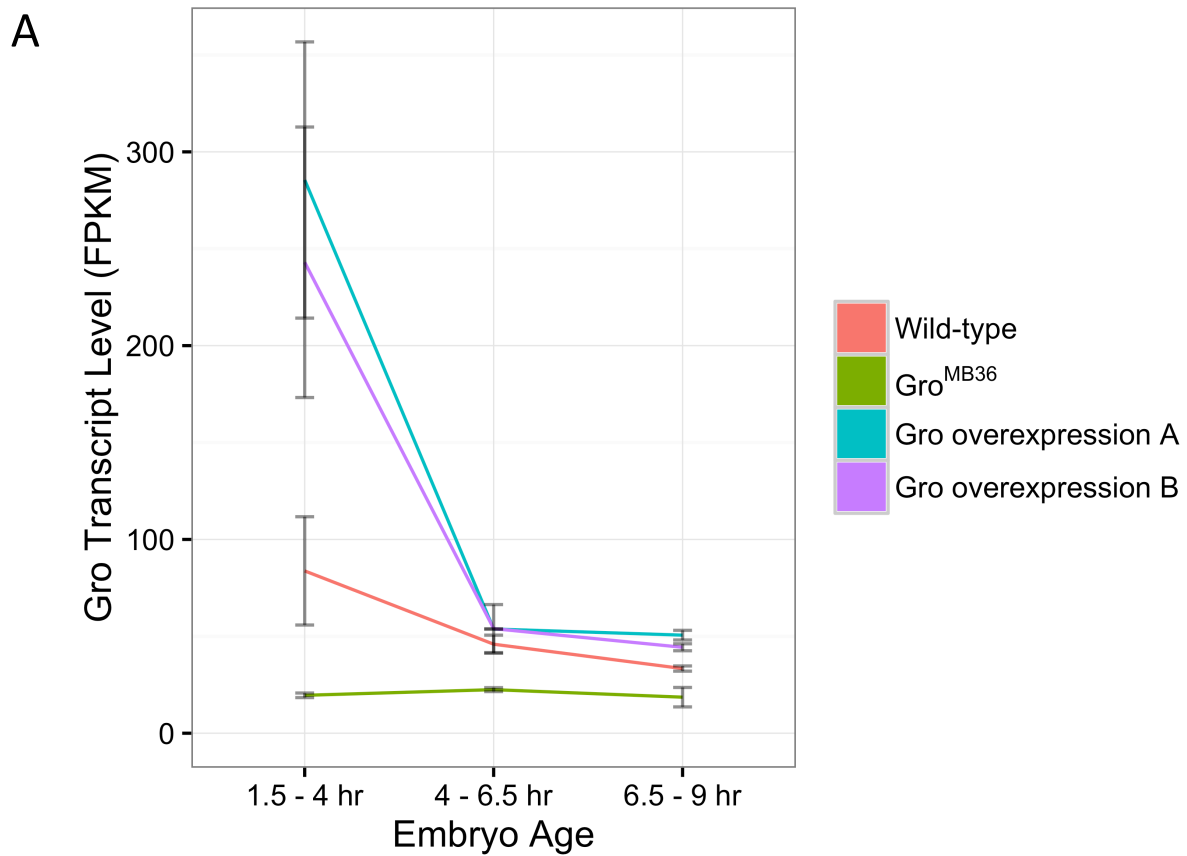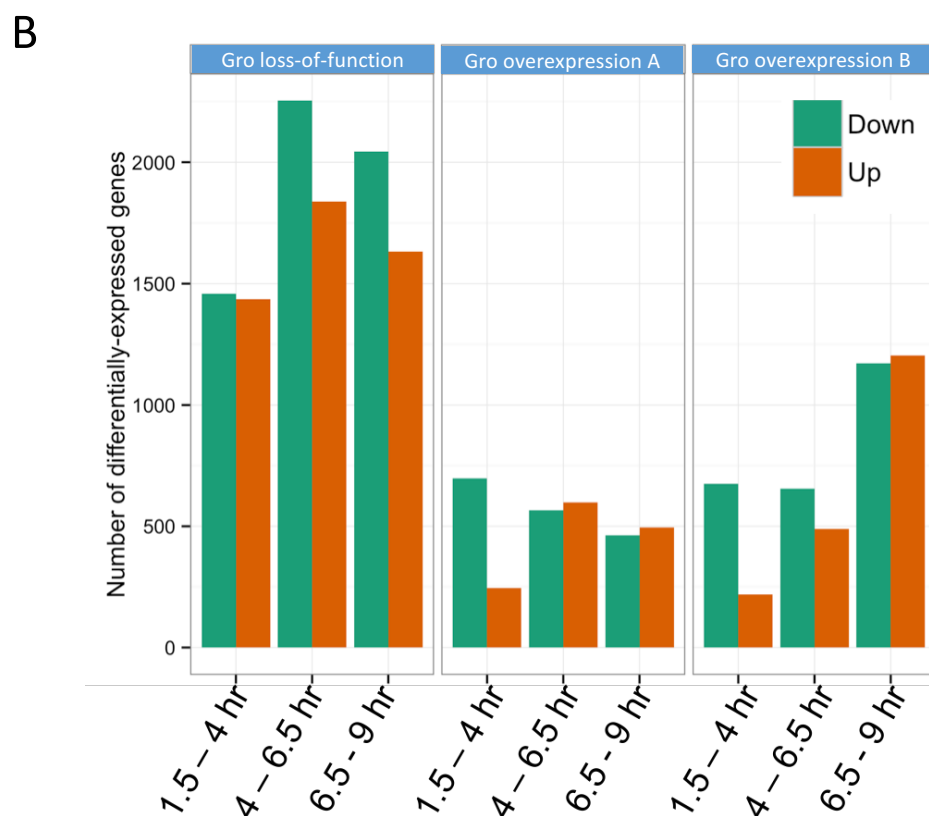

Figure S2

Supplement: Additional file 4: Figure S2. — Fractions of genes showing altered expression in Gro overexpression and Gro LOF embryos. (A) Normalized Gro transcript expression levels were calculated at each timepoint. (B) Maternal Gro deficiency results in a large proportion (>10%) of expressed genes becoming misregulated in the Drosophila embryo across all time points. Overexpression of Gro results in a smaller but still significant alteration of the embryonic transcription profile. (PDF 451 kb) [file 12864_2017_3589_MOESM4_ESM.pdf]

A

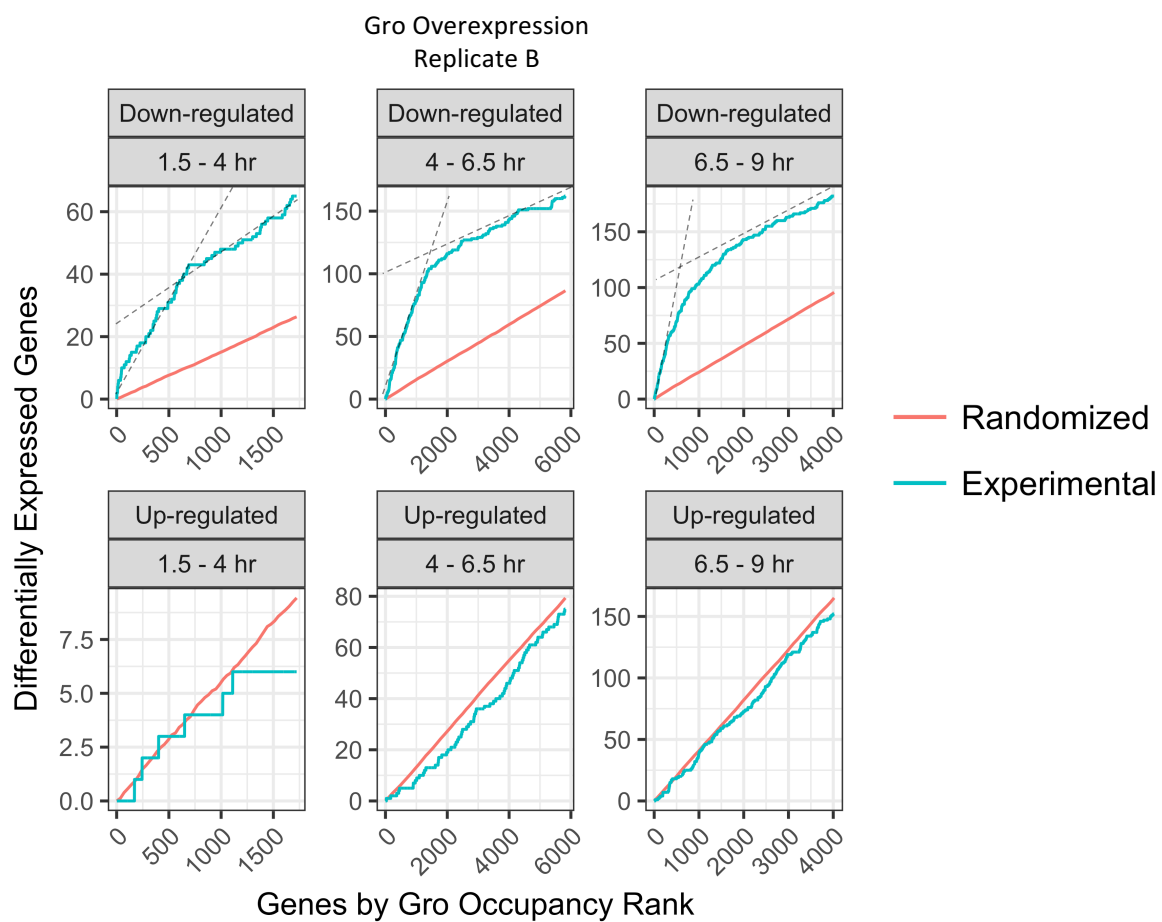

B

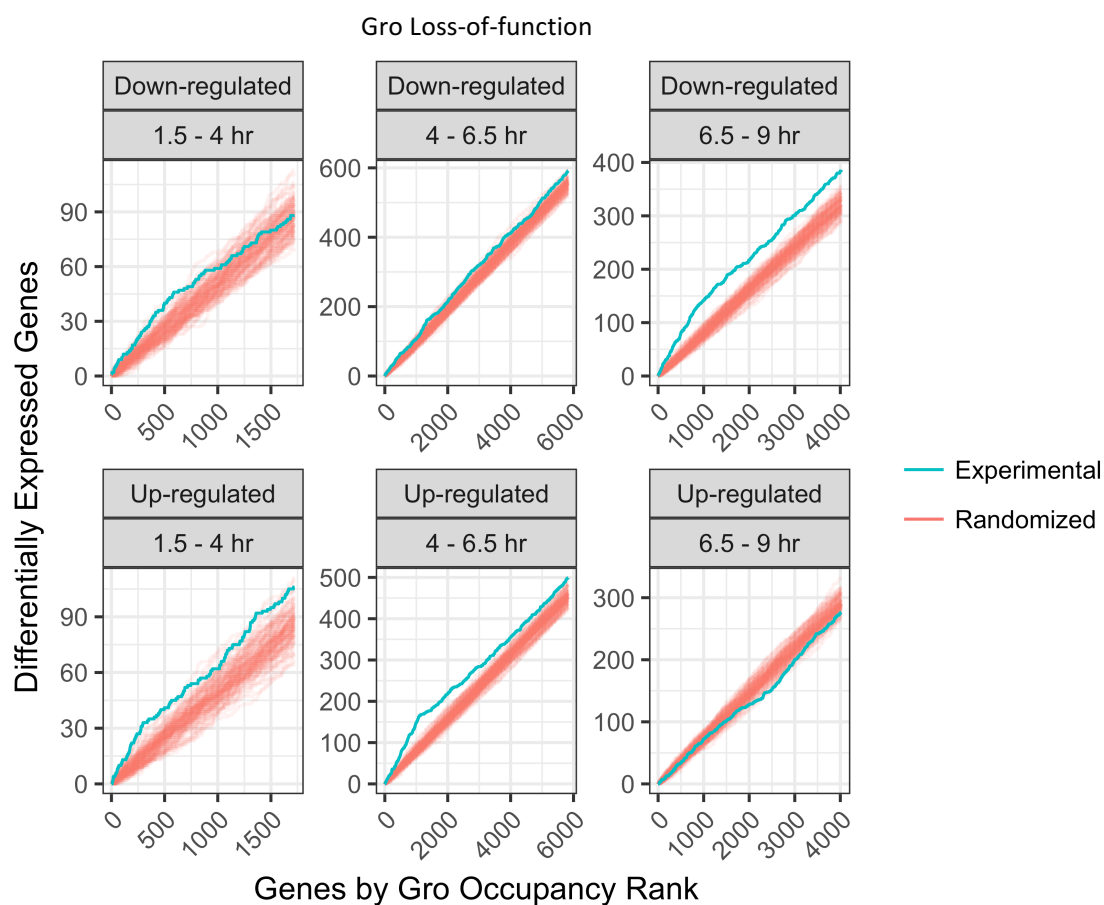

Figure S3

Supplement: Additional file 5: Figure S3. — Numbers of differentially expressed genes in Gro overexpression and loss-of-function embryos as a function of Gro occupancy score. These graphs are similar to those in Fig. 5, except that they incorporate the RNA-seq data from (A) Gro overexpression line B, and (B) the Gro maternal loss-of-function embryos. For (A) each “Randomized” curve is the average (linear interpolation) of 100 trials with 100 sets of randomly generated genes. Randomized gene sets contained identical numbers of up- and down-regulated genes as found in the original data. For (B), rather than averaging the results from the random gene sets, the results for each random gene set are plotted as a separate curve. (PDF 2313 kb) [file 12864_2017_3589_MOESM5_ESM.pdf]

A

### All Gro Targets

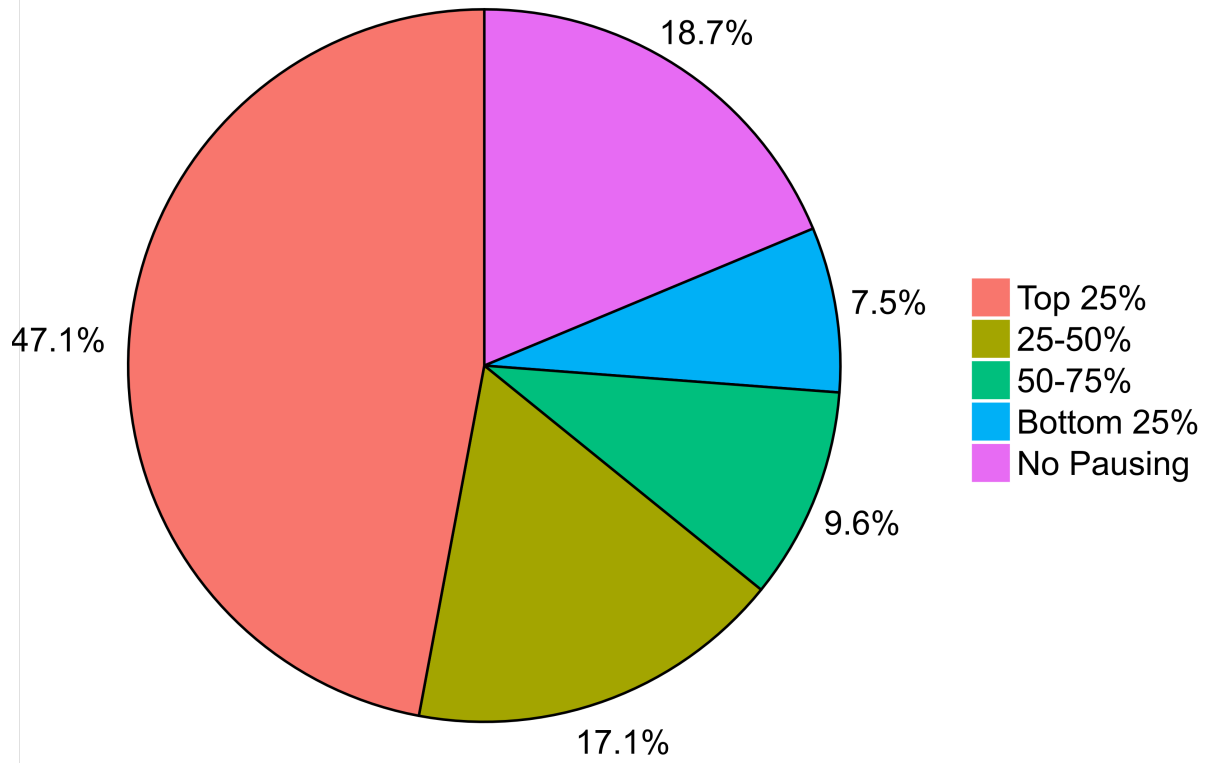

B

### 1.5 – 4 hr Gro Targets

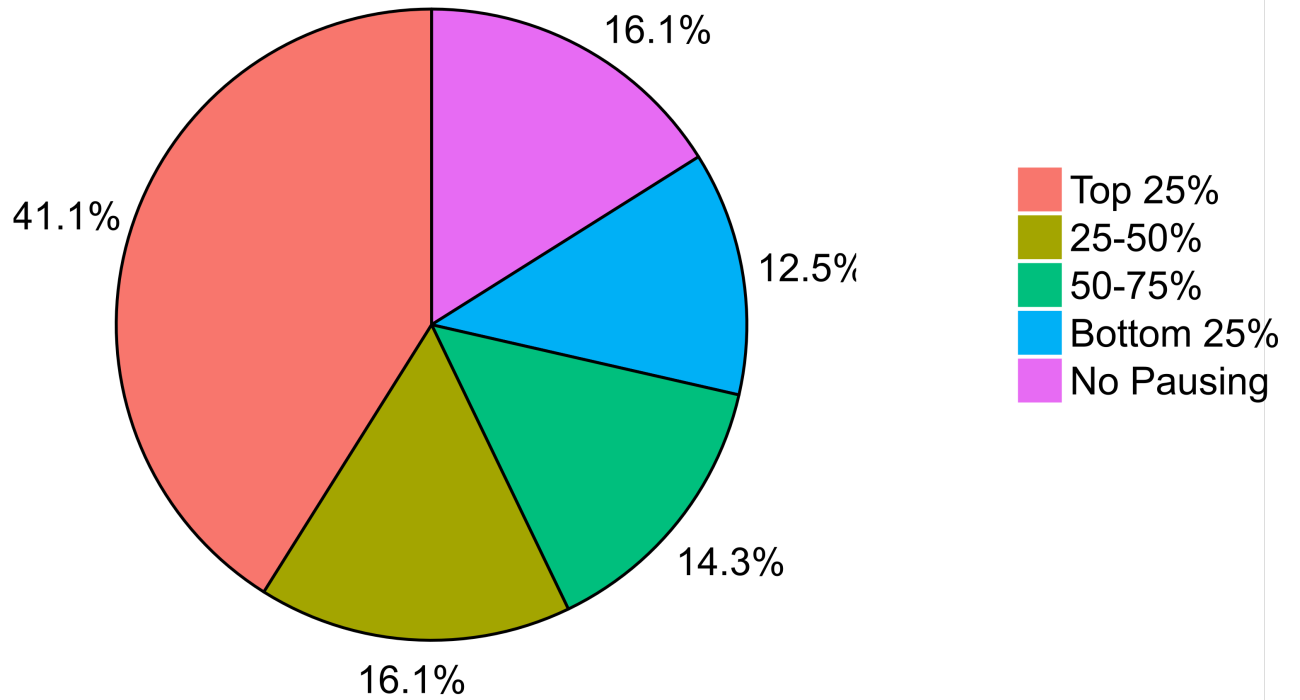

Figure S5

Supplement: Additional file 7: Figure S5. — Gro-regulated genes are enriched for genes with high pausing index based on GRO-seq analysis. The prevalence of Pol II promotor-proximal pausing in predicted Gro targets was calculated across all timepoints (1.5–9 hrs, A) or in the first timepoint only (1.5–4 hrs, B). An index of promotor-proximal PolII pausing was obtained from GRO-seq data generated in 2–2.5 hr old wild-type embryos (see Saunders et al., 2013 for details [97]). The resulting genes were split into quartiles based on pausing index and compared to predicted Gro targets. Approximately 41% of predicted Gro targets in 1.5–4 hr embryos were found in the upper quartile, indicating a strong association between Gro regulation and polymerase pausing. This enrichment for paused genes is highly significant, with a p-value of < 10−10 by a Wilcoxon rank sum test. (PDF 758 kb) [file 12864_2017_3589_MOESM7_ESM.pdf]

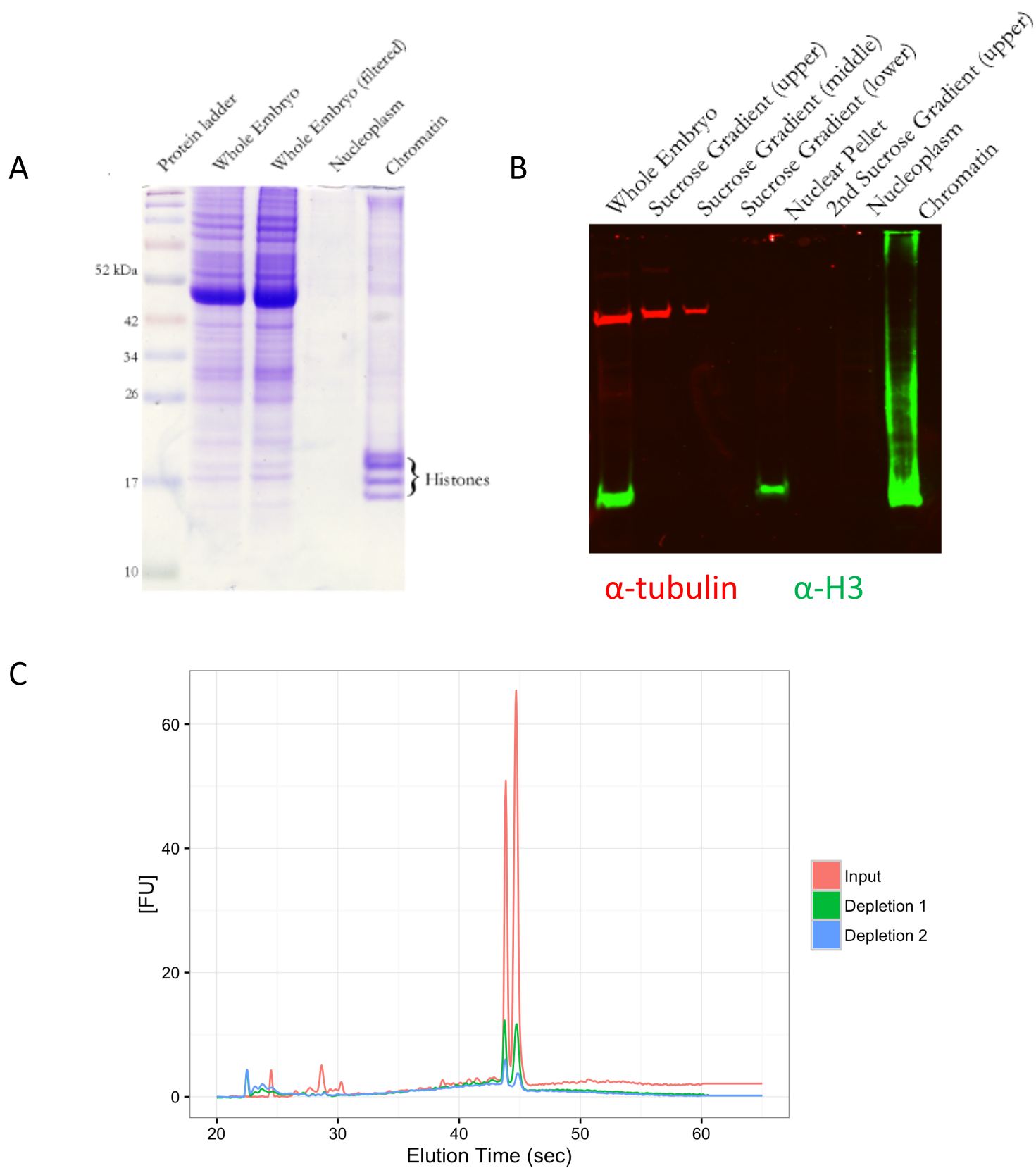

Figure S4

Supplement: Additional file 8: Figure S4. — Validation of enrichment of chromatin-associated RNA from total embryonic RNA. (A) Protein components of embryo fractions utilized for RNA-seq were visualized with SDS-PAGE. Isolated chromatin was enriched for multiple bands in the 15 to 19 kDa range consistent with histone core proteins. (B) Immulobloting reveals the lack of a cytoplasmic marker (tubulin) in the nucleoplasmic and chromatin fractions, as well as the presence of histone H3 in the chromatin fraction. (C) As chromatin-associated RNA is largely non-polyadenylated, poly(A) + affinity techniques commonly utilized to isolate mRNA from the much larger pools of non-coding transcripts could not be utilized. An affinity depletion protocol was instead used to remove the major D. melanogaster rRNA transcripts as well as other non-coding RNAs prior to high-throughput sequencing. Depletion of the two major rRNA species (28 s and 18 s) was confirmed via Agilent Bioanalyzer RNA profiles. Large rRNA peaks in the input indicate the RNA pool underwent minimal degradation during fractionation and purification. (PDF 427 kb) [file 12864_2017_3589_MOESM8_ESM.pdf]
