# Supplementary material for: Mechanisms of Groucho-mediated repression revealed by genome-wide analysis of Groucho binding and activity
Source: BMC Genomics. 2017 Feb 28;18:215. doi: 10.1186/s12864-017-3589-6 (PMC5331681; doi:10.1186/s12864-017-3589-6)
Supplement: Additional file 9: Table S4. — Overlap of sequenced reads from chromatin-associated and total poly(A) + RNA-seq libraries. (DOCX 44 kb) [file 12864_2017_3589_MOESM9_ESM.docx]

**Table S4**. Overlap of sequenced reads from chromatin-associated and total poly(A)+ RNA-seq libraries

|  |  | **% of Library Reads** | | | |
| --- | --- | --- | --- | --- | --- |
|  | **Embryo Age** | **Exon** | **Intron** | **5' UTR** | **3' UTR** |
| **Chromatin-associated** | 1.5 - 4 hr | 82% | 19% | 4% | 4% |
|  | 4 - 6.5 hr | 55% | 47% | 8% | 5% |
|  | 6.5 - 9 hr | 64% | 39% | 7% | 6% |
| **poly(A)+** | 1.5 - 4 hr | 77% | 10% | 4% | 16% |
|  | 4 - 6.5 hr | 75% | 14% | 3% | 15% |
|  | 6.5 - 9 hr | 79% | 13% | 4% | 17% |
