# Supplementary material for: Mechanisms of Groucho-mediated repression revealed by genome-wide analysis of Groucho binding and activity
Source: BMC Genomics. 2017 Feb 28;18:215. doi: 10.1186/s12864-017-3589-6 (PMC5331681; doi:10.1186/s12864-017-3589-6)
Supplement: Additional file 10: Table S5. — Low and High abundance transcripts from chromatin-associated RNA-seq. (DOCX 112 kb) [file 12864_2017_3589_MOESM10_ESM.docx]

**Table S5**: Low and High abundance transcripts from chromatin-associated RNA-seq

| Lower Relative Abundance | | |
| --- | --- | --- |
| **BDGP Term Enrichment** | **p-value** | **# genes** |
| maternal | 3.09E-86 | 1541 |
| ubiquitous | 7.16E-45 | 847 |
| anterior midgut primordium | 6.21E-29 | 505 |
| posterior midgut primordium | 2.40E-26 | 514 |
| trunk mesoderm primordium | 1.80E-20 | 411 |
| anterior endoderm primordium | 3.51E-15 | 292 |
| embryonic midgut | 5.09E-15 | 587 |
| posterior endoderm primordium | 5.20E-14 | 299 |
| dorsal prothoracic pharyngeal muscle | 1.13E-12 | 220 |
| head mesoderm primordium P2 | 1.76E-09 | 253 |
| embryonic/larval muscle system | 3.69E-09 | 277 |
| head mesoderm primordium | 8.15E-09 | 153 |
| posterior endoderm primordium P2 | 2.19E-08 | 218 |
| anterior endoderm anlage | 6.20E-08 | 205 |
| faint ubiquitous | 1.26E-07 | 437 |
| Higher Relative Abundance | | |
| **BDGP Term Enrichment** | **p-value** | **# genes** |
| ventral nerve cord | 1.19E-24 | 452 |
| ventral epidermis primordium | 5.92E-24 | 159 |
| dorsal ectoderm primordium | 9.17E-23 | 152 |
| dorsal ectoderm anlage in statu nascendi | 3.07E-22 | 137 |
| ventral ectoderm anlage in statu nascendi | 1.25E-20 | 125 |
| embryonic brain | 1.14E-19 | 431 |
| dorsal epidermis primordium | 2.17E-19 | 167 |
| procephalic ectoderm anlage in statu nascendi | 5.27E-19 | 123 |
| ventral ectoderm primordium P2 | 2.40E-18 | 169 |
| ventral ectoderm primordium | 7.18E-17 | 139 |
| gap | 6.36E-13 | 56 |
| embryonic ventral epidermis | 1.43E-12 | 245 |
| procephalic ectoderm anlage | 1.44E-12 | 140 |
| embryonic dorsal epidermis | 2.56E-12 | 266 |
| tracheal primordium | 7.27E-12 | 90 |
